# Monitoring changes in the prevalence of the Beijing lineage of *Mycobacterium tuberculosis* in Ecuador: 2018 and 2023

**DOI:** 10.3389/fpubh.2026.1967382

**Published:** 2026-10-27

**Authors:** Ángel Sebastián Rodríguez-Pazmiño, Lilian Paola Pozo-Estupiñán, Melany Patricia Cedeño-Vásquez, Irina Trejo Cedeño, Greta Cárdenas-Franco, Andrea Belen Salcedo Maldonado, Natalia Jiménez-Pizarro, Javier Hermoso de Mendoza, Solon Alberto Orlando, Greta Franco-Sotomayor, Miguel Ángel Garcia-Bereguiain

**Affiliations:** 1 One Health Research Group, Universidad de Las Américas, Quito, Ecuador; 2 Universidad Católica Santiago de Guayaquil, Guayaquil, Ecuador; 3 Universidad Ecotec, Samborondón, Ecuador; 4 Departamento de Sanidad Animal, Facultad de Veterinaria, Universidad de Extremadura, Cáceres, Spain; 5 Instituto Nacional de Salud Pública e Investigación, Guayaquil, Ecuador

**Keywords:** Beijing lineage, Ecuador, MIRU-VNTR, molecular epidemiology, *Mycobacterium tuberculosis*, SNP-PCR, whole genome sequencing

## Abstract

**Background:**

The *Mycobacterium tuberculosis* Beijing sublineage (Lineage 2) is a major global health concern due to its association with increased transmissibility, hypervirulence, and multidrug resistance (MDR). Although it is dominant in East Asia, its prevalence in South America is generally low, except in some locations in Peru and Colombia, where local prevalences reach up to 16.4 and 15.6%, respectively. Previous studies in Ecuador have reported a very low prevalence (0.43–1.6%), suggesting a lack of active transmission clusters. This study aims to update the molecular epidemiology of the Beijing sublineage in Ecuador by analyzing recent isolates from 2018 and 2023.

**Methods:**

We analyzed 699 clinical *M. tuberculosis* isolates collected in 2018 and 2023 using stepwise genotyping. We first screened isolates by allele-specific SNP-PCR targeting the Rv2154724 (G526A) marker, then confirmed results with 24-locus MIRU-VNTR and spoligotyping. We conducted whole-genome sequencing (WGS) on the Beijing-positive isolate to refine lineage assignment and drug-resistance profiling. We assessed genetic relatedness through Minimum Spanning Tree (MST) analysis, incorporating all 12 previously reported *M. tuberculosis* Beijing isolates in Ecuador.

**Results:**

Only one isolate from the 384 strains from 2018 (0.26%) was identified as belonging to the *M. tuberculosis* Beijing sublineage, while no Beijing isolate was detected among the 315 strains from 2023 by SNP-PCR screening. This isolate, collected in 2018 from Machala (El Oro province), was confirmed as Beijing by SNP-PCR and MIRU-24 and additionally as sublineage 2.2.1 by WGS. MST analysis revealed moderate genetic diversity and no active transmission clusters or temporal groupings among Ecuadorian Beijing strains. The identified *M. tuberculosis* Beijing isolate exhibited a genotypically predicted MDR profile with resistance to rifampicin, isoniazid, ethambutol, and streptomycin.

**Conclusion:**

The *M. tuberculosis* Beijing sublineage remains ultra-low in prevalence in Ecuador, with no evidence of consolidated community transmission, even in high-risk border regions. These findings challenge the global consensus on the hyper-adaptability of Beijing strains in South America. Furthermore, our results underscore the limitations of spoligotyping and highlight the need to strengthen and expand genomic surveillance (including WGS) to accurately monitor high-risk *M. tuberculosis* lineages.

## 1 Introduction

Tuberculosis (TB) remains a critical global health challenge, with approximately 10.7 million new cases and 1.2 million deaths in 2024 (1). In this context, molecular epidemiology is a fundamental tool for identifying transmission clusters and understanding the pathogen’s dynamics (2). Among the various genotypes of *Mycobacterium tuberculosis* (MTB), the Beijing family (Lineage 2) is of particular concern due to its selective advantages, including increased transmissibility, hypervirulence, and a strong association with multidrug-resistant (MDR) and extensively drug-resistant (XDR) strains (3, 4). Recent studies indicate that the global success of this family is largely driven by “modern” sublineages (specifically clades L2.3.4 to L2.3.6), which exhibit significantly higher transmissibility and higher genome mutation rates than their “ancient” counterparts (5, 6).

While the Beijing lineage is dominant in East Asia, its prevalence in South America has historically remained below 5% (7). However, Ecuador shares open borders with Peru and Colombia, countries where some local studies have documented significantly higher prevalences of 16.4 and 15.6%, respectively (8, 9). Additionally, in Southwestern Colombia, specific “modern” Beijing clusters (SIT190) have been strongly linked to localized MDR-TB outbreaks (9). Despite this proximity and intense regional trade, previous countrywide screenings in Ecuador have documented a very low prevalence of the Beijing sublineage, ranging from 1.6% in 2014–2016 using MIRU-VNTR to as low as 0.43% in subsequent larger assessments (10, 11). These findings have challenged the consensus regarding the hyper-adaptability of Beijing strains within the Ecuadorian population, as isolates have shown high genetic diversity and a notable lack of active transmission clusters, even in potentially high-risk transmission setings such as the border provinces of El Oro and Esmeraldas (12, 13).

To accurately monitor potential shifts in these dynamics, high-resolution genotyping is essential. Single-nucleotide polymorphism (SNP) PCR is a rapid, cost-effective first-line screening tool for detecting the Beijing-specific Rv2154724 (G526A) marker (14). For deeper characterization, 24-locus Mycobacterial Interspersed Repetitive Unit-Variable Number Tandem Repeat (MIRU-VNTR) typing provides the necessary discriminatory power to identify clonal complexes and confirm lineage assignments (15). Additionally, spoligotyping remains a standard method for identifying the characteristic genomic footprints (spacers 35 to 43) of the Beijing family (16).

This study aimed to evaluate the current status of the MTB Beijing sublineage in Ecuador by analyzing clinical isolates from 2018 and 2023. Using a stepwise genotyping approach that includes SNP-PCR screening, followed by MIRU-VNTR and spoligotyping for lineage confirmation, this study aims to identify changes in prevalence and transmission patterns compared with previous reports. Additionally, we performed whole-genome sequencing (WGS) on the Beijing-positive isolate to provide high-resolution confirmation of lineage assignment and characterize its genotypic drug-resistance profile.

## 2 Methodology

### 2.1 Sample collection and study design

This study analyzed clinical MTB isolates collected in Ecuador in 2018 and 2023 and preserved at the *Laboratorio de Referencia Nacional para Micobacterias* (CRN-Micobacterias) of the *Instituto Nacional de Investigación en Salud Pública* (INSPI) in Guayaquil, Ecuador. This laboratory-based study used archived clinical isolates rather than probabilistic sampling; therefore, we did not perform a formal sample-size calculation. The analyzed isolates represent a complete census of all MTB isolates available and adequately preserved at INSPI for the two study years, with no additional selection criteria applied beyond availability. During data curation, we identified and removed two back-up aliquots that INSPI inadvertently supplied as separate tubes but that corresponded to samples already included in the study; after this curation, the final dataset comprised 699 isolates (384 from 2018 and 315 from 2023).

As the national mycobacterial reference center, INSPI receives isolates referred from across the country, and referral and culture procedures followed the same standardized protocols in both study years. All isolates were obtained from newly diagnosed tuberculosis patients, and we included only one isolate per patient, so no patient was represented by more than one isolate within a given year or across the two study years. We did not select isolates consecutively or randomly; we included all available preserved isolates, with no selection based on drug-resistance profile. The collection consisted almost entirely of pulmonary tuberculosis cases (696/699; 99.6%), with only three extrapulmonary cases (0.4%), all from 2023. We classified sample type according to WHO criteria based on the site of disease; isolates without an explicit sample-type annotation in the INSPI databases were considered pulmonary, as respiratory specimens (e.g., sputum) are frequently registered without a specified type, whereas non-respiratory specimens are consistently annotated.

We selected 2018 and 2023 as the earliest and most recent years for which cultured isolates remained available and adequately preserved at INSPI. Isolates from 2017 were no longer available because storage and infrastructure constraints (including recurrent power outages) affected the collection; given the budget limitations inherent to this type of study in Ecuador, we prioritized the two extreme years in the available range to maximize the temporal window for comparison.

At this facility, we cultured isolates using the Kudoh–Ogawa method, adapted from the protocol described by Franco-Sotomayor et al. (17). INSPI confirmed the identification of the *M. tuberculosis* complex by immunochromatographic detection of the MPT64 antigen. All procedures involving viable *M. tuberculosis* were performed at the INSPI reference laboratory under biosafety level 2 enhanced (BSL-2+) conditions, in accordance with WHO tuberculosis laboratory biosafety guidelines. We harvested mycobacterial colonies, resuspended them in Tris–EDTA (TE) buffer (10 mM Tris–HCl, 1 mM EDTA, pH 8.0), and inactivated them at 95 °C for 15 min. After heat inactivation, we centrifuged samples at 10,000 × g for 5 min and used the supernatant directly as a template for molecular assays, as described by Aldous et al. (18). We then transported processed samples to the research laboratories at the Universidad de Las Américas (Quito, Ecuador), where we stored them at −20 °C until further analysis.

The study followed a stepwise genotyping approach in which each method served a distinct and complementary purpose. First, all isolates were screened for the Beijing lineage using allele-specific SNP-PCR, selected as a rapid, low-cost, and high-throughput first-line tool suitable for the large number of isolates analyzed. Isolates identified as Beijing-positive were then characterized by 24-locus MIRU-VNTR, which confirms lineage assignment and enables assessment of genetic relatedness among strains (used here for the minimum spanning tree analysis), and by spoligotyping as a complementary genotyping method. Finally, whole-genome sequencing was performed on the Beijing-positive isolate to provide high-resolution confirmation of the sublineage and to infer the genotypic drug-resistance profile. We adopted this multi-method strategy because no single technique could efficiently screen and resolve lineage with high confidence.

### 2.2 SNP-PCR for Beijing lineage identification

We performed allele-specific PCR targeting a lineage-defining single-nucleotide polymorphism (SNP) to identify isolates belonging to the Beijing lineage, following the protocol described by Mora-Pinargote et al. (11). Each reaction used a final volume of 12 μL containing 6 μL of GoTaq® Green Master Mix (1×), 0.6 μL of each primer (final concentration 0.5 μM), 2.4 μL of genomic DNA, and 2.4 μL of nuclease-free water. Genomic DNA was obtained by boiling preparations from cultured isolates (as described in section 2.1). We quantified a random subset of samples by spectrophotometry (NanoDrop), consistently yielding concentrations above 10 ng/μL; because the two allele-specific reactions used the same template, successful amplification served as an intrinsic confirmation of sufficient amplifiable DNA for each sample. Two independent reactions were performed per isolate, sharing a common reverse primer (5’-CTGCACACCGATGAATTCGC-3’) and differing only in the 3’-terminal nucleotide of the forward primer, which coincides with the Rv2154724 (G526A) polymorphic site: one forward primer ends in A (5’-CTGCTGCGCCCAGGAA-3’) and the other ends in G (5’-CTGCTGCGCCCAGGAG-3’). Discrimination between Beijing and non-Beijing isolates is based on the differential amplification efficiency between the two reactions, determined by the number of 3′ mismatches against the template: for Beijing strains, the A-ending forward primer shows a single mismatch and therefore amplifies more efficiently, whereas for non-Beijing strains the G-ending primer amplifies more efficiently.

Thermal cycling conditions consisted of an initial denaturation at 95 °C for 3 min, followed by 35 cycles of denaturation at 95 °C for 30 s, annealing at 58 °C for 30 s, and extension at 72 °C for 30 s, with a final extension at 72 °C for 5 min. All SNP-PCR runs included a positive control (a Beijing strain previously characterized by Castro-Rodríguez et al. and maintained in our laboratory collection) and a no-template negative control.

Although a formal analytical sensitivity value has not been established for this allele-specific SNP-PCR assay, previous studies in Ecuador have validated and applied it for large-scale Beijing screening (10, 11). To minimize misclassification, we further confirmed all SNP-PCR–positive isolates by MIRU-VNTR, spoligotyping, and whole-genome sequencing.

### 2.3 PCR product analysis and interpretation

We resolved PCR products by electrophoresis on 2% agarose gels and visualized them with SYBR Safe under ultraviolet illumination. We included positive and negative controls. We assigned Beijing lineage based on the relative intensity of amplification bands, following the criteria described by Garzón-Chávez et al. (10) and Mora-Pinargote et al. (11). We considered an isolate Beijing-positive when the reaction with the A-ending forward primer produced a more intense band than the reaction with the G-ending primer. We performed band-intensity analysis using Image Lab software. Gel electrophoresis images for all SNP-PCR reactions are provided in Supplementary Figure S1.

### 2.4 MIRU-VNTR genotyping and lineage assignment

We further characterized isolates identified as Beijing-positive by SNP-PCR using MIRU-VNTR genotyping with a 24-locus scheme. We separated PCR products on 2% agarose gels and determined the number of tandem repeats at each locus by amplicon size. We generated numerical allelic profiles for each isolate and analyzed them with the MIRU-VNTRplus platform^1^ to assign lineages according to established classification criteria (19).

### 2.5 Minimum spanning tree analysis

We performed minimum spanning tree (MST) analyses using MIRU-VNTR 24-loci profiles from the Ecuadorian Beijing isolate identified in the present study and from previously published reports. We incorporated additional international Beijing reference isolates from different geographic regions to provide a global phylogeographic context. Allelic profiles were organized into a tab-delimited dataset containing isolate identifiers and corresponding MIRU-24 locus values. We incorporated epidemiological metadata, including country of origin, province, year of isolation, and antibiotic resistance status, as auxiliary data for visualization. The MIRU-24 codes used in these analyses are presented in the Supplementary Table S1.

MSTs were generated using the goeBURST algorithm implemented in PHYLOViZ Online (20).^2^ Genetic relationships among isolates were inferred based on allelic differences across the MIRU-24 loci. Nodes in the network represented unique MIRU-VNTR profiles, while edges corresponded to the number of allelic variations between connected isolates. Distinct visualizations were produced by coloring nodes according to epidemiological variables, including geographic origin, year of isolation, and antibiotic resistance status, allowing exploratory assessment of clustering patterns and potential transmission relationships.

Because MIRU-VNTR is based on allelic profile comparisons rather than whole-genome sequence data, we interpreted the resulting MSTs as representations of genetic relatedness and epidemiological clustering rather than definitive phylogenetic reconstructions.

### 2.6 Spoligotyping analysis

Spoligotyping was performed to confirm lineage assignment by assessing the presence or absence of 43 spacer sequences within the direct repeat (DR) region of the *M. tuberculosis* genome, following the method described by Kamerbeek et al. (21). Briefly, DR loci were amplified by PCR using primers DRa and DRb. Amplified products were biotin-labeled and hybridized to a membrane containing immobilized oligonucleotides corresponding to each spacer sequence. After hybridization, membranes were washed and incubated with streptavidin–peroxidase conjugate (Roche, United States), followed by chemiluminescent detection using the Amersham ECL system (GE Healthcare, United Kingdom).

Signals were visualized on Hyperfilm ECL (Merck, United States) using X-ray exposure, then developed and fixed in a darkroom. The resulting hybridization patterns were recorded in binary and octal formats for interpretation in genotyping. Spoligotype patterns were compared with the international SpolDB4 database to classify lineages (22). This analysis was conducted at the research laboratories of the Facultad de Veterinaria, Universidad de Extremadura (Cáceres, Spain).

### 2.7 Whole genome sequencing and genomic analysis

We performed WGS on the isolate identified as Beijing by SNP-PCR to confirm lineage assignment and characterize drug-resistance-associated mutations. We extracted genomic DNA using the PureLink Genomic DNA Mini Kit (Invitrogen, Thermo Fisher Scientific, United States) and performed paired-end sequencing on an Illumina MiSeq system (2 × 150 bp) at Biosequence (Quito, Ecuador). The raw paired-end reads (R1 and R2) were used directly as input for TBProfiler (version 6.6.5), which performs internal read trimming and alignment to the *M. tuberculosis* H37Rv reference genome (NC_000962.3) prior to variant calling. We obtained lineage and sublineage classification and genotypic drug-resistance prediction using TBProfiler’s default mutation database (tbdb; commit 0a909094), which incorporates lineage-defining SNPs and resistance-associated variants cataloged by the World Health Organization. The median genome coverage for the sequenced isolate was 35.8×. We inferred genotypic resistance profiles from mutations in genes associated with resistance to first- and second-line anti-tuberculosis drugs, following the interpretation framework implemented in TBProfiler.

### 2.8 WGS data availability

We deposited the whole-genome sequencing data generated for the Beijing-positive isolate in this study in the NCBI Sequence Read Archive (SRA) under BioProject accession PRJNA1524051 (BioSample SAMN62949040; SRA run SRR40532610).

## 3 Results

### 3.1 Prevalence and molecular identification of MTB Beijing sublineage

We analyzed 699 MTB isolates: 384 from 2018 and 315 from 2023. Only one MTB strain belonged to the MTB Beijing sublineage. This isolate was collected in 2018 from Machala, El Oro province, representing a prevalence of 0.26% that year, while no Beijing isolate was detected in 2023 by SNP-PCR screening. This isolate (sample ID: Mach 1,277) was initially classified as Beijing by SNP-PCR (Supplementary Figure S1, gel image 15) and then by MIRU-24 analysis (code: 234232252643425152353823), while spoligotyping did not match any registered pattern in the SITVIT/SpolDB4 database (assigned as “not identified”/orphan), and the MIRU-VNTRplus database assigned it to the Delhi/CAS lineage (octal code: 677777600060770). However, WGS analysis using TB-Profiler confirmed its classification as Beijing lineage 2.2.1.

### 3.2 WGS drug resistance analysis

WGS analysis also identified the isolate as MDR-TB, carrying canonical resistance-associated mutations in genes encoding targets of first-line anti-tuberculosis drugs. These included *rpoB* p. His445Tyr, associated with rifampicin resistance; *katG* p. Ser315Thr, associated with high-level isoniazid resistance; *embB* p. Met306Val, associated with ethambutol resistance; and *rpsL* p. Lys43Arg, associated with streptomycin resistance. No mutations associated with resistance to fluoroquinolones, second-line injectable agents, bedaquiline, linezolid, delamanid, or pretomanid were detected, suggesting that the isolate belongs to the MDR-TB category without evidence of pre-XDR or XDR-associated resistance markers.

### 3.3 Minimum spanning tree analysis of Ecuadorian Beijing isolates

We constructed an MST based on 24-locus MIRU-VNTR profiles from all MTB Beijing isolates previously reported in Ecuador (2014 to 2016), along with the isolate identified in the present study in 2018 (ECU_Beijing_13). The analysis included 13 unique MIRU-VNTR profiles, with their geographical distribution shown in Figure 1.

**Figure 1:**
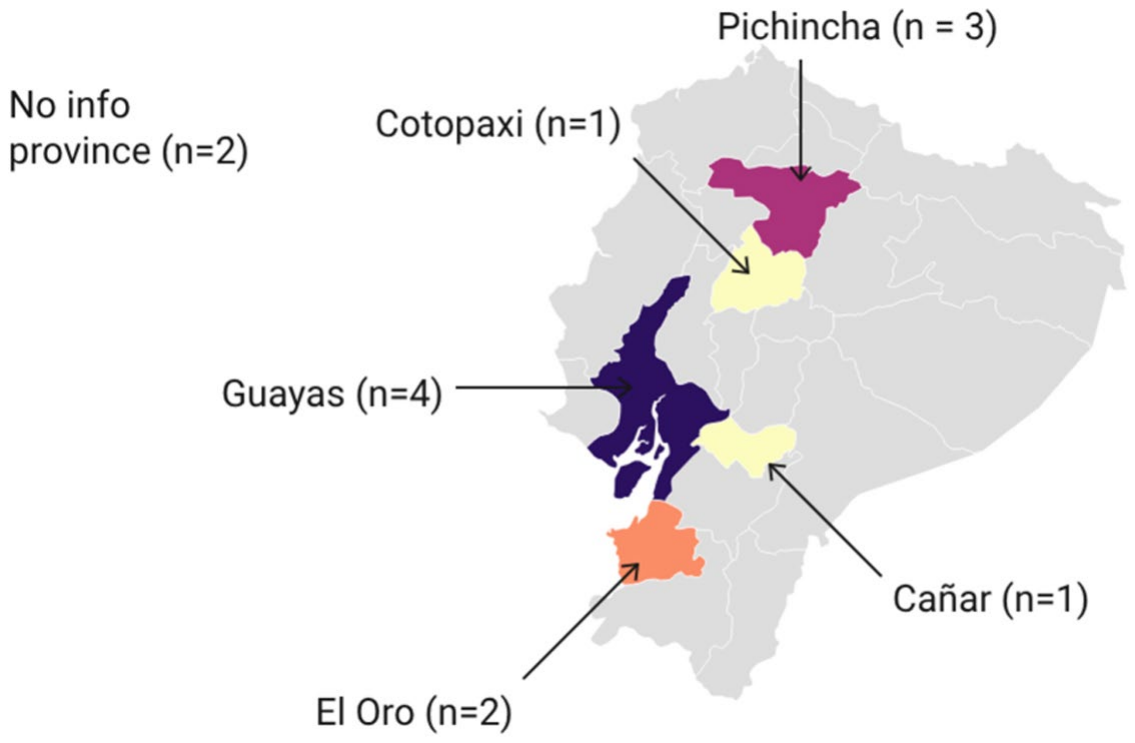
Provincial distribution of *Mycobacterium tuberculosis* Beijing strains in Ecuador. Distribution based on a compilation of 13 isolates previously described in the literature, plus one clinical isolate collected in Machala (El Oro, 2018) in this study.

The resulting network revealed moderate genetic diversity among Ecuadorian Beijing isolates, with several closely related profiles connected through a central node (ECU_Beijing_04) and multiple peripheral branches (Figure 2). No completely isolated clusters were observed, and most isolates were connected by relatively short allelic distances. When the MST was visualized by province of origin, isolates from different geographic regions were distributed throughout the network, without forming province-specific clusters (Figure 2a). Similarly, visualization according to drug-resistance status showed that resistant and susceptible isolates were distributed across different branches of the tree rather than concentrated within a single genetic cluster (Figure 2b). Temporal visualization also revealed related MIRU-VNTR profiles across different years of isolation, with no clear temporal clustering pattern (Figure 2c).

**Figure 2:**
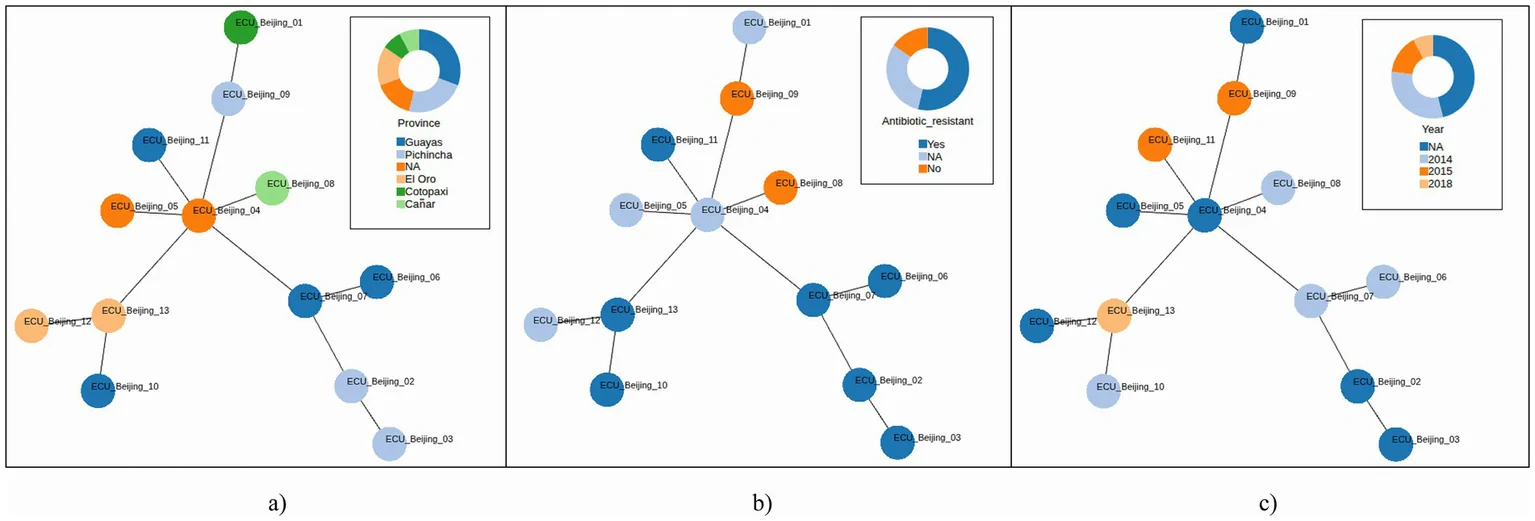
Minimum spanning tree analysis of MTB Beijing strains isolated in Ecuador. The sample from our study corresponds to ECU_Beijing_13. **(a)** MST colored by the province location of the sample; **(b)** colored by antibiotic resistance (presence = yes, absence = no); **(c)** colored by year of collection. NA = no information.

To place the Ecuadorian isolates in a global context, another MST was generated using MIRU-24 profiles from Beijing strains reported in multiple countries, including Ecuador, China, Russia, Georgia, Kazakhstan, Moldova, Ukraine, and Colombia (Figure 3). Ecuadorian isolates were distributed across several regions of the network rather than forming a single country-specific cluster. Several Ecuadorian profiles were positioned near isolates from Eurasian countries, particularly Russia, Georgia, Kazakhstan, and China (Figure 3a). Visualization by drug-resistance status showed that resistant isolates were broadly distributed throughout the network and not restricted to a single branch or lineage (Figure 3b). Both resistant and susceptible isolates were observed among the major clusters identified in the international dataset.

**Figure 3:**
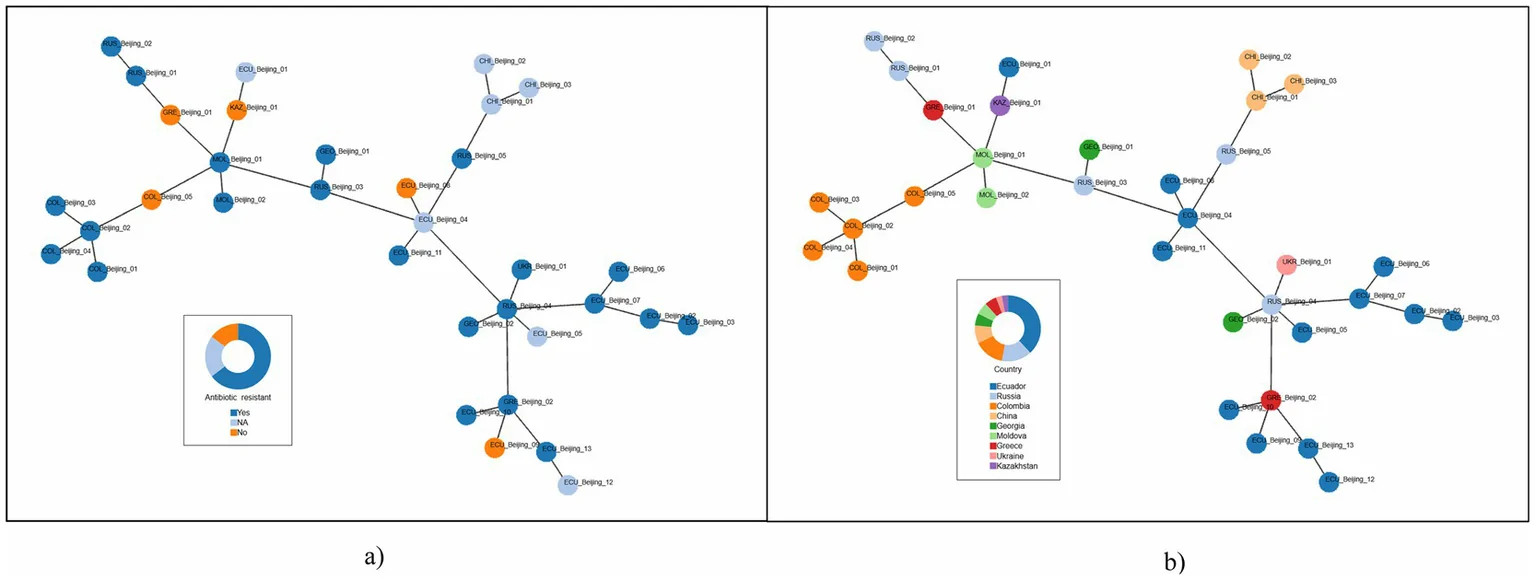
Minimum spanning tree analysis of MTB Beijing strains isolated worldwide including Ecuador. The sample from our study corresponding to ECU_Beijing_13. **(a)** MST colored by antibiotic resistance (presence = yes, absence = no); **(b)** colored by country.

## 4 Discussion

These findings confirm that the MTB Beijing sublineage (Lineage 2) remains very rare in Ecuador, even in more recent clinical isolates from 2018 and 2023. Our reported prevalence of 0.26% (1/384) for 2018 and 0% for 2023 (no detected by SNP-PCR screening) is consistent with the very low prevalence of 0.43% identified in a massive 2014–2016 countrywide screening (11). These results further challenge the global consensus on the hyper-adaptability and rapid expansion of Beijing strains in South America (6). While this lineage accounts for nearly a quarter of all TB cases globally and dominates East Asia (23), its presence in most South American countries (with notable exceptions in certain areas of Peru and Colombia) has historically been low (7).

The population structure of *M. tuberculosis* in Ecuador has been characterized in previous studies, which consistently report that Lineage 4 (Euro-American) is by far the most prevalent lineage in the country, with the LAM family predominating, whereas Lineage 2 (Beijing) remains rare (10, 12). Although the present study was designed as a targeted screening for the Beijing lineage and did not determine the complete lineage distribution of the analyzed isolates, our finding of an ultra-low Beijing prevalence is fully consistent with this established population structure, in which Lineage 4 dominates and Beijing circulates only sporadically.

The unique geographic position of Ecuador, which shares open borders with Peru (MTB Beijing prevalence up to 16.4%) and Colombia (MTB Beijing prevalence up to 15.6%), makes the continued rarity of Beijing strains particularly striking (10, 11). In our study, the only Beijing isolate was detected in Machala (El Oro province), a border region characterized by high TB incidence and intense cross-border migration. However, our finding aligns with recent research by Castro-Rodriguez et al. (12), which analyzed isolates from the same province and identified only a single Beijing strain (1.8%), concluding that TB dynamics in this border area are likely driven by relapses of latent cases rather than the active transmission of hypervirulent Beijing clusters from neighboring Peru (Castro-Rodriguez, León-Ordóñez, et al., 2024).

MST analysis of the 13 available MTB Beijing cultures isolated in Ecuador reveals moderate genetic diversity and no active transmission clusters. The absence of province-specific clustering or temporal groupings suggests that the MTB Beijing strains circulating in Ecuador represent sporadic, independent introductions or reactivations of long-standing latent infections rather than a consolidated community outbreak (Castro-Rodriguez, León-Ordóñez, et al., 2024 (10);). This is further supported by the international context of MTB Beijing strains isolated in Ecuador displayed in Figure 3, showing no clustering of Ecuador with MTB Beijing strains from neighbor countries like Peru or Colombia, but with MTB Beijing Eurasian lineages from countries such as Russia, China, and Georgia. This pattern may reflect historical waves of migration from East Asia to South America that occurred before the antibiotic era (24).

Methodologically, the misidentification of our isolate by spoligotyping—which incorrectly assigned it to the Delhi/CAS lineage—is highly significant. This phenomenon highlights the limitations of spoligotyping, which is based on repetitive elements prone to convergent evolution (3, 14). In contrast, allele-specific SNP-PCR and WGS targeting stable markers such as Rv2154724 (G526A) proved essential for accurate lineage assignment (14). Our results advocate strengthening and expanding genomic surveillance to avoid misclassifying high-risk lineages.

This study has several limitations. First, we identified only a single Beijing isolate across the entire sample set; while this clearly establishes the rarity of the Beijing lineage in Ecuador, it precludes population-level inference about its genetic diversity, clustering, or transmission dynamics. Second, screening was based on allele-specific SNP-PCR, for which a formal analytical sensitivity value has not been determined; SNP-PCR–negative isolates (including all 2023 samples) were not further analyzed by whole-genome sequencing, and we therefore cannot formally exclude the presence of Beijing strains below the detection conditions of the screening assay. Nonetheless, this is a validated screening method, and we confirmed all SNP-PCR–positive isolates by MIRU-VNTR, spoligotyping, and whole-genome sequencing, minimizing the risk of misclassification. Third, the resistance profile of the Beijing isolate was inferred genotypically from whole-genome sequencing (TBProfiler) and was not confirmed by phenotypic drug-susceptibility testing, which was not available in this study; although genotypic prediction shows high concordance with phenotypic results for the first-line drugs that define the MDR phenotype (rifampicin and isoniazid) (23, 25, 26), phenotypic confirmation would further strengthen the resistance characterization. Fourth, the analyzed isolates correspond to culture-positive strains referred to and preserved at the national reference laboratory, predominantly from Coastal provinces, and may therefore not fully represent the national distribution of TB cases in Ecuador. Finally, as a minor methodological note, we did not quantify DNA for every sample; however, the two-reaction design of the allele-specific assay ensured that successful amplification confirmed sufficient template in each case.

Finally, the identified 2018 isolate showed a genotypically predicted MDR-TB profile (resistant to rifampicin, isoniazid, ethambutol, and streptomycin), underscoring the ongoing threat posed by MTB Beijing strains. Historically, MTB Beijing isolates have shown a high association with multidrug resistance, particularly in Guayaquil (10, 11). Although we did not find evidence of active transmission, the hypervirulence and higher genome mutation rates characteristic of “modern” Beijing sublineages (like 2.2.1) mean that these strains could rapidly adapt to escape host immune responses or acquire further resistance under inadequate treatment regimens (6, 27). In this sense, these type of recurrent monitoring studies are recommended for an active tracking of emerging MDR MTB lineages.

In conclusion, while the prevalence of the MTB Beijing lineage in Ecuador remains extremely low for years 2018 and 2023, the presence of an MDR-TB strain in a high-risk border province like El Oro endorses continued molecular monitoring. Strengthening MTB lineages surveillance is vital for detecting potential shifts in transmission dynamics that could arise from evolving regional migration crises.

## Data Availability

The original contributions presented in the study are included in the article/Supplementary material, further inquiries can be directed to the corresponding author/s.

## Footnotes

1
https://www.miru-vntrplus.org/

2
https://online.phyloviz.net
