# Supplementary material for: Monitoring changes in the prevalence of the Beijing lineage of Mycobacterium tuberculosis in Ecuador: 2018 and 2023
Source: Front Public Health. 2026 Oct 27;14:1967382. doi: 10.3389/fpubh.2026.1967382 (PMC13748251; doi:10.3389/fpubh.2026.1967382)

Supplementary Material — Gel electrophoresis images of the Beijing SNP-PCR screening

Supplementary Figure S1. Agarose gel electrophoresis images of the allele-specific SNP-PCR screening for the Beijing lineage across all analyzed *M. tuberculosis* isolates. The document comprises 27 gel images. In each gel, samples are organized into two sets of reactions per isolate: one performed with the forward primer whose 3′-terminal nucleotide corresponds to the Beijing-associated allele (labeled "SNP [reaction number]") and the other with the forward primer corresponding to the non-Beijing allele (labeled "NO SNP [reaction number]"), sharing a common reverse primer. All reactions yield an amplicon of the same expected size; Beijing versus non-Beijing status is determined by the relative band intensity between the two reactions rather than by band presence or absence. Each gel includes a molecular-weight ladder ("L") and a no-template control (NTC); most gels also include a Beijing positive control ("C. Beijing") consisting of a strain previously confirmed as Beijing by MIRU-VNTR and whole-genome sequencing. The single Beijing-positive isolate identified in this study (MYCO_025_Ago25; reaction no. 281) is shown in gel image 15, where the "SNP 281" reaction shows markedly higher amplification intensity than the "NO SNP 281" reaction, consistent with the Beijing genotype.

Gel image 1

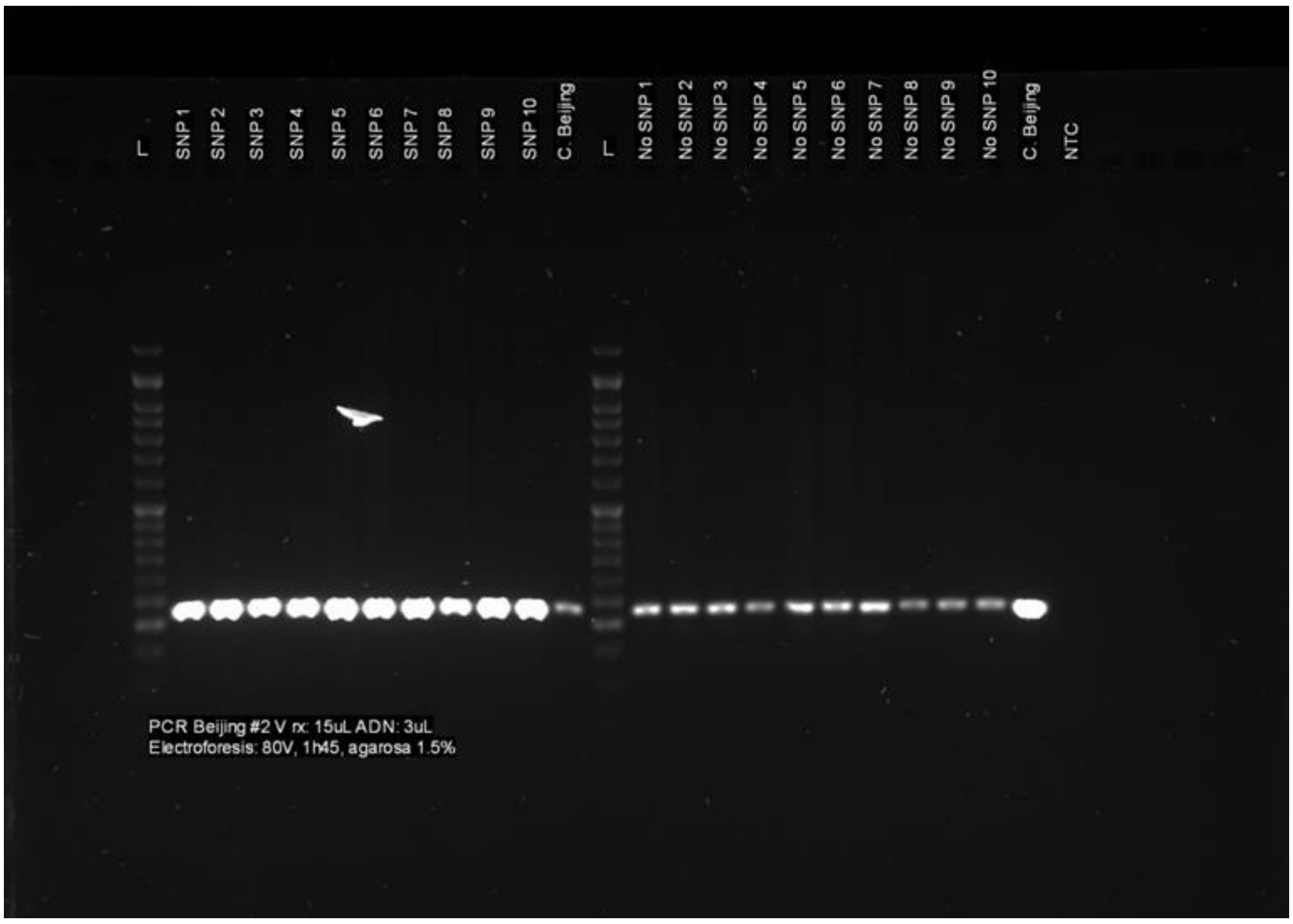

Gel image 2

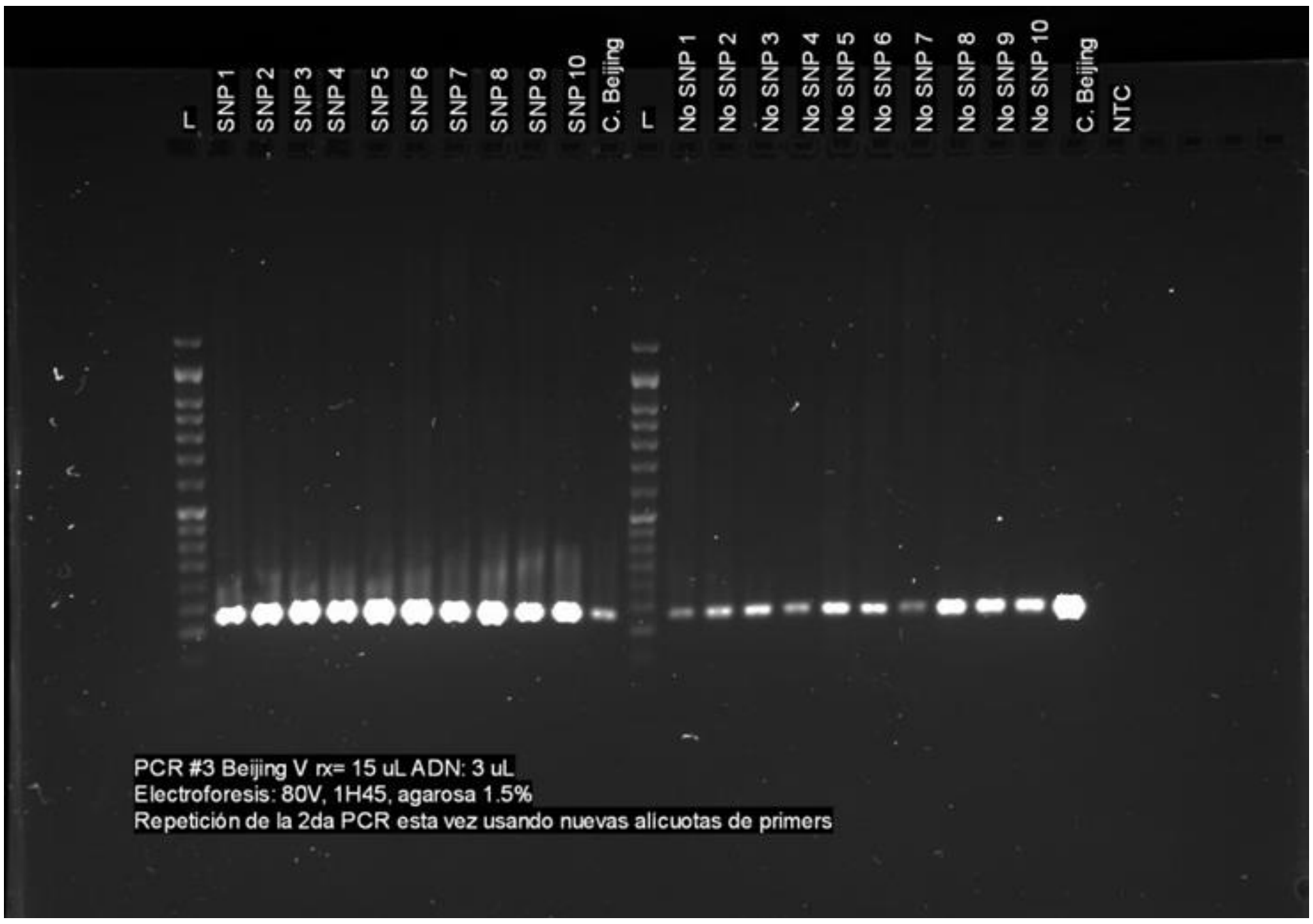
L
SNP 1
SNP 2
SNP 3
SNP 4
SNP 5
SNP 6
SNP 7
SNP 8
SNP 9
SNP 10
C. Beijing
L
No SNP 1
No SNP 2
No SNP 3
No SNP 4
No SNP 5
No SNP 6
No SNP 7
No SNP 8
No SNP 9
No SNP 10
C. Beijing
NTC
PCR #3 Beijing V rx= 15 uL ADN: 3 uL
Electroforesis: 80V, 1H45, agarosa 1.5%
Repetición de la 2da PCR esta vez usando nuevas alicuotas de primers

Gel image 3

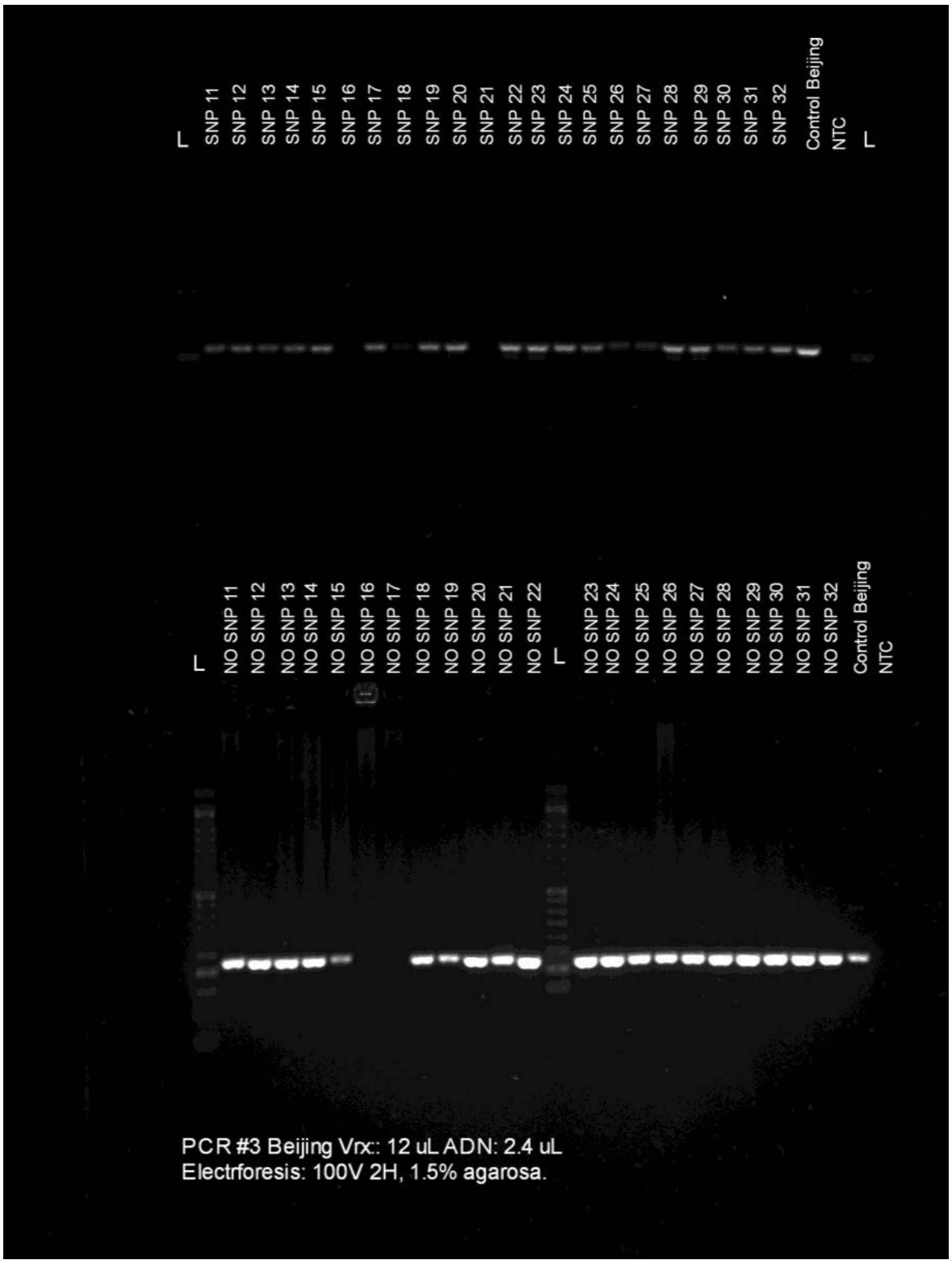
L
SNP 11
SNP 12
SNP 13
SNP 14
SNP 15
SNP 16
SNP 17
SNP 18
SNP 19
SNP 20
SNP 21
SNP 22
SNP 23
SNP 24
SNP 25
SNP 26
SNP 27
SNP 28
SNP 29
SNP 30
SNP 31
SNP 32
Control Beijing
NTC
L
L
NO SNP 11
NO SNP 12
NO SNP 13
NO SNP 14
NO SNP 15
NO SNP 16
NO SNP 17
NO SNP 18
NO SNP 19
NO SNP 20
NO SNP 21
NO SNP 22
L
NO SNP 23
NO SNP 24
NO SNP 25
NO SNP 26
NO SNP 27
NO SNP 28
NO SNP 29
NO SNP 30
NO SNP 31
NO SNP 32
Control Beijing
NTC
PCR #3 Beijing Vrx:: 12 uL ADN: 2.4 uL
Electrforesis: 100V 2H, 1.5% agarosa.

Gel image 4

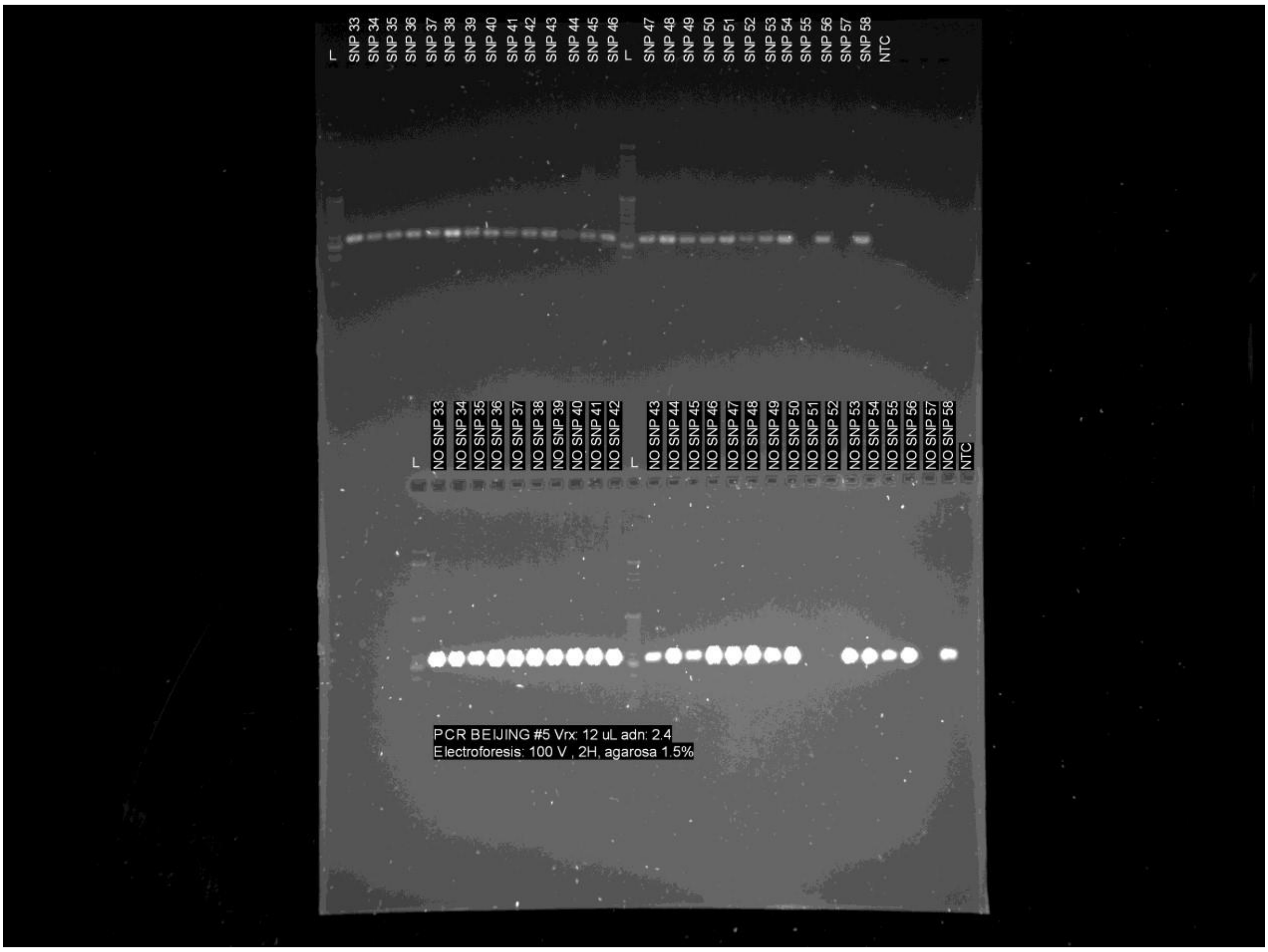

Gel image 5

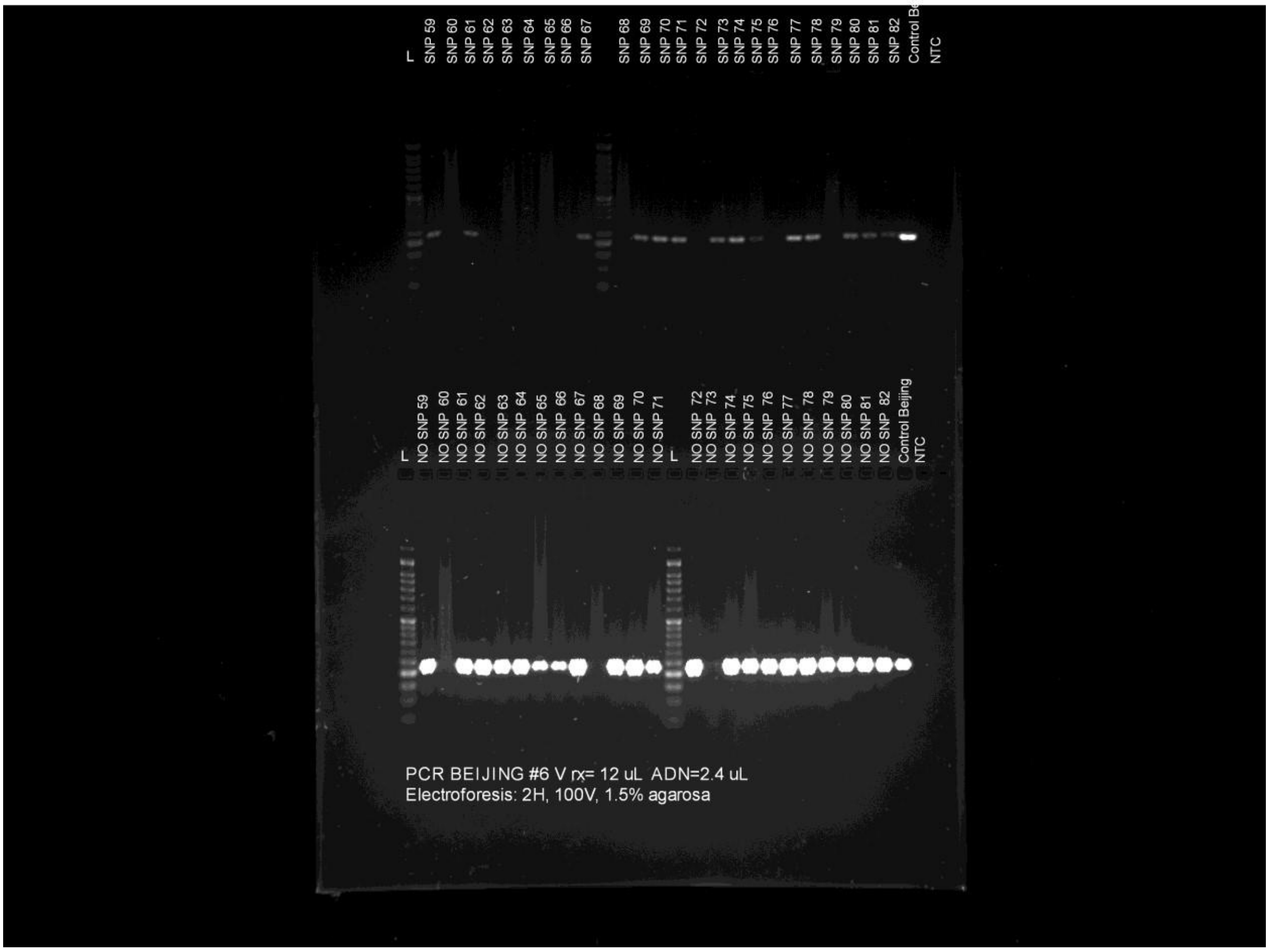

Gel image 6

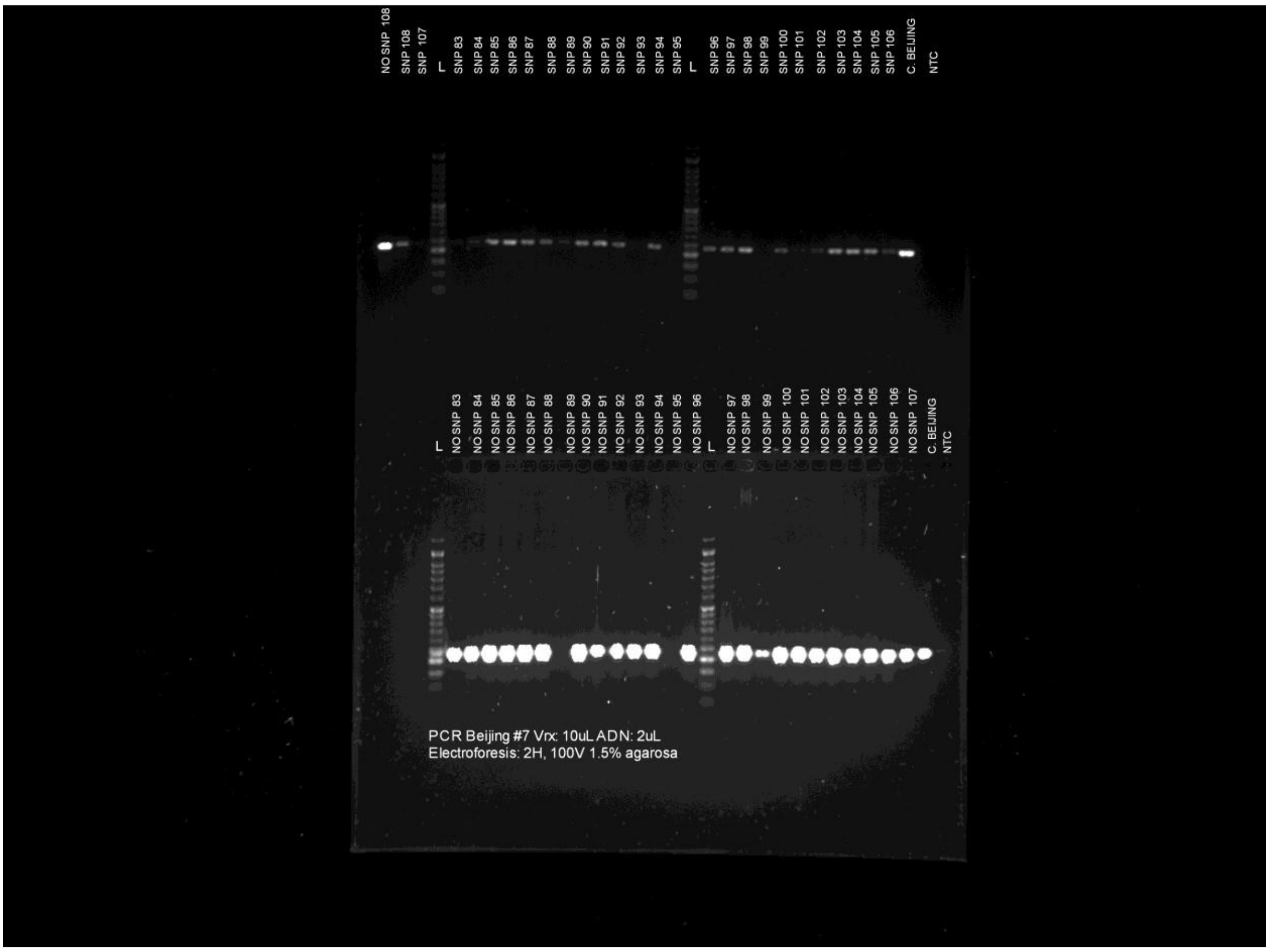

Gel image 7

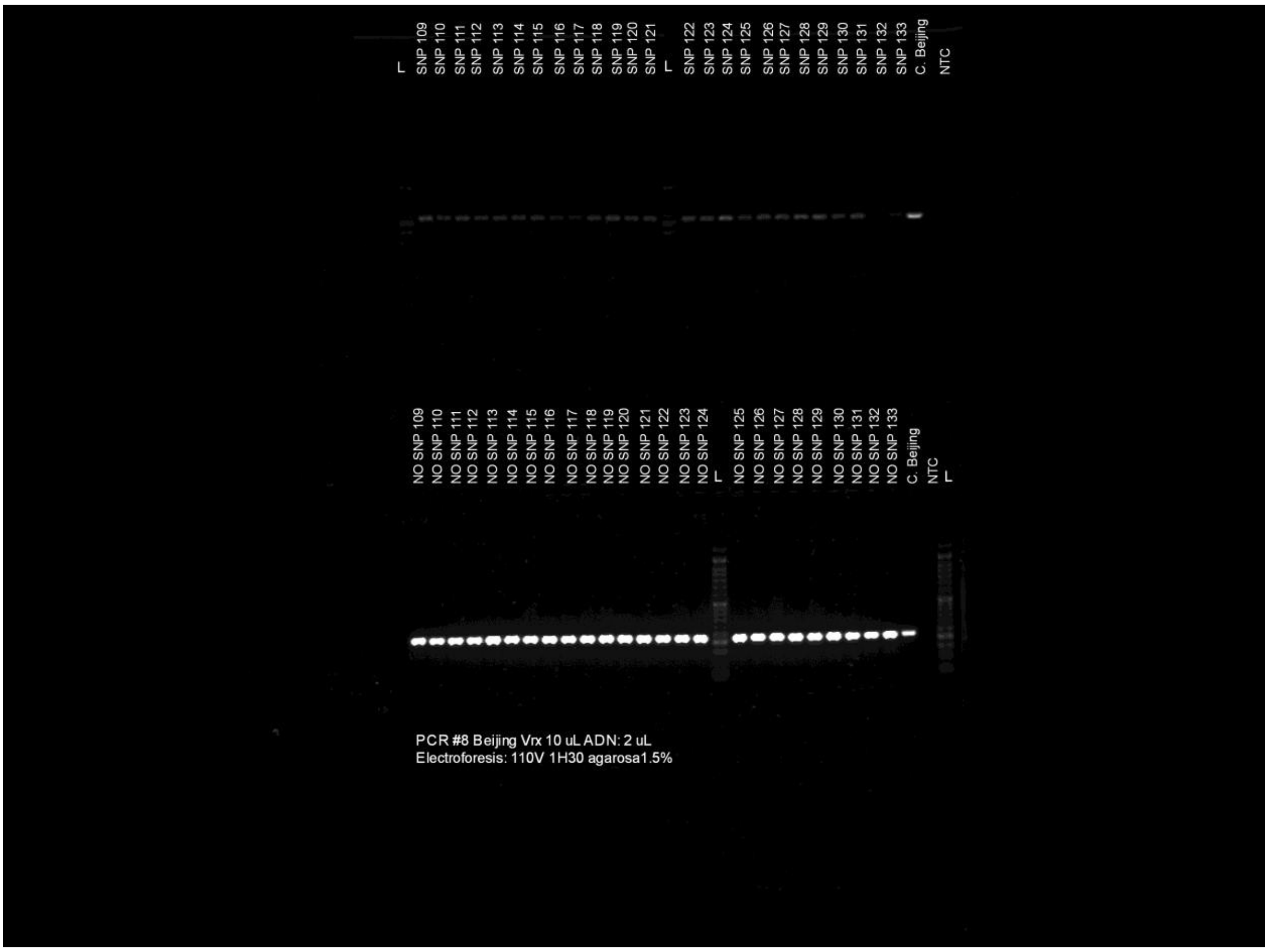

Gel image 8

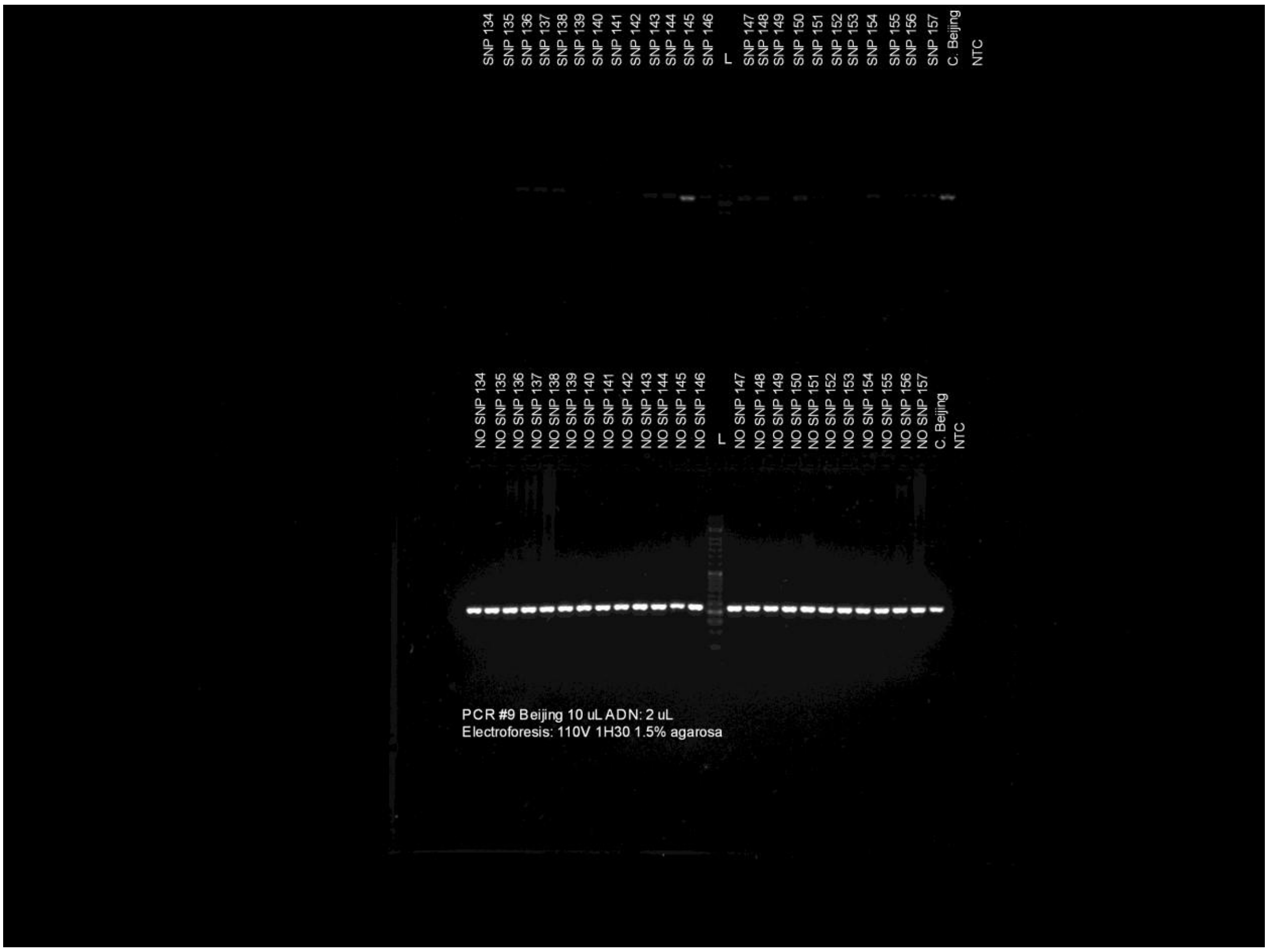

Gel image 9

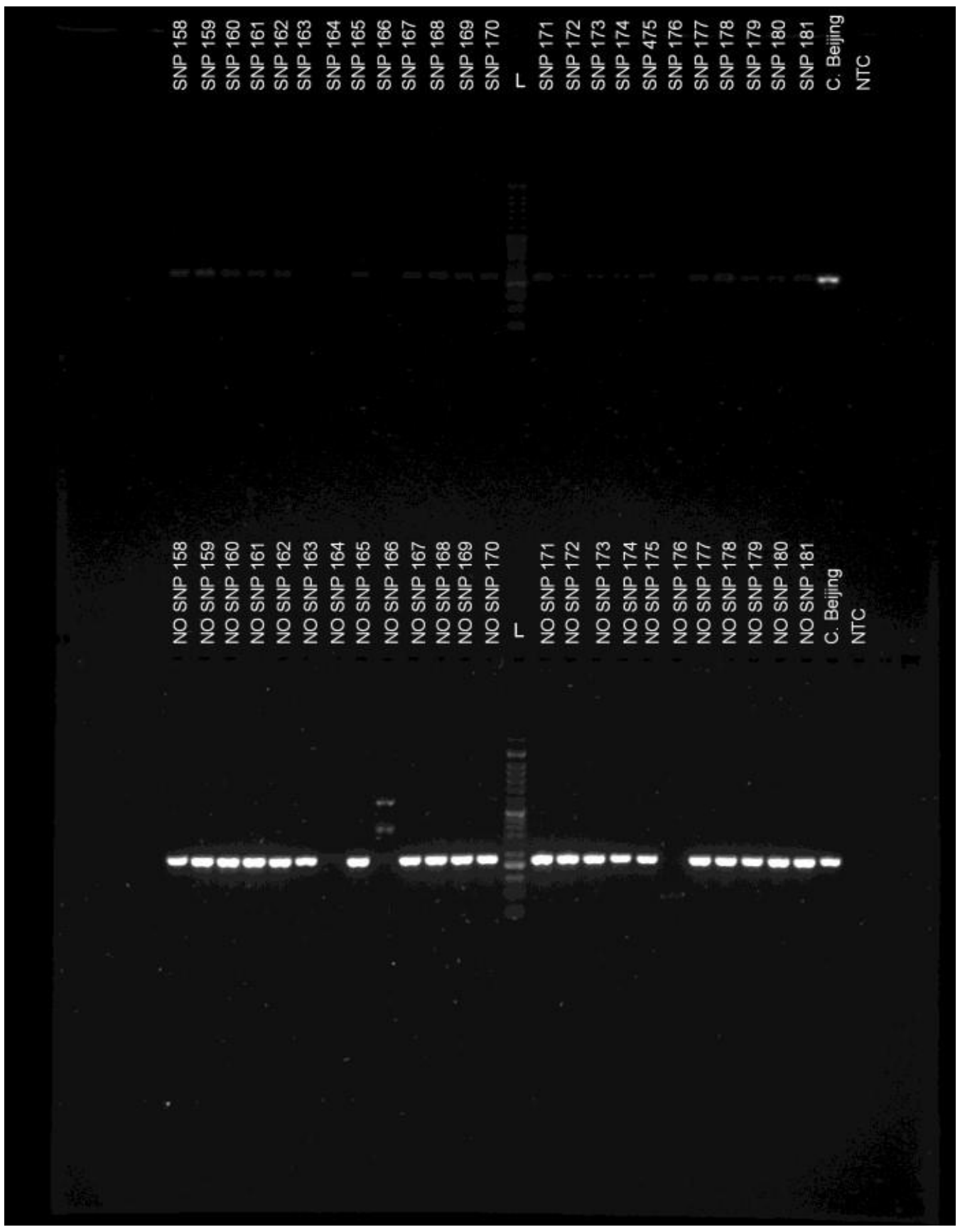

Gel image 10

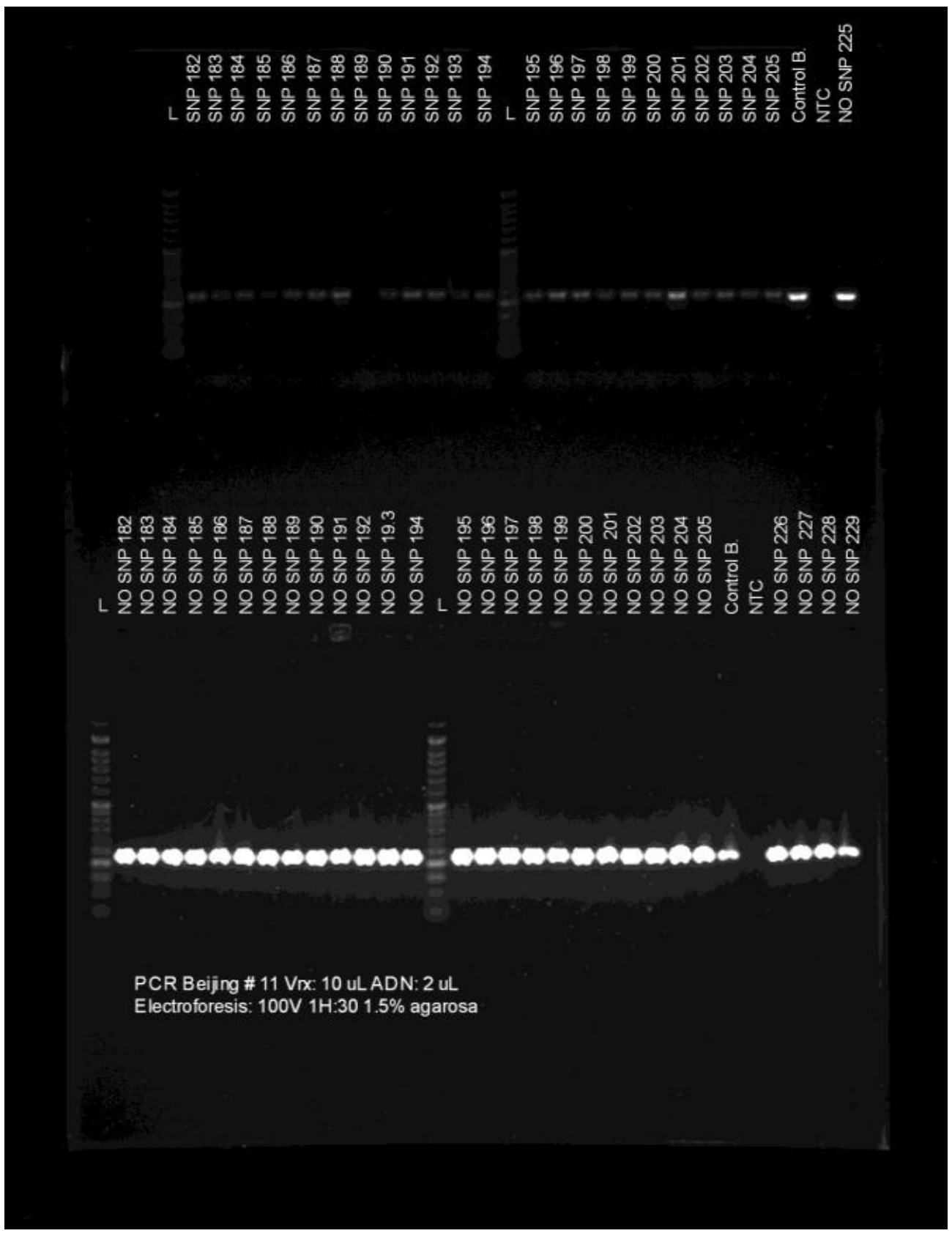

Gel image 11

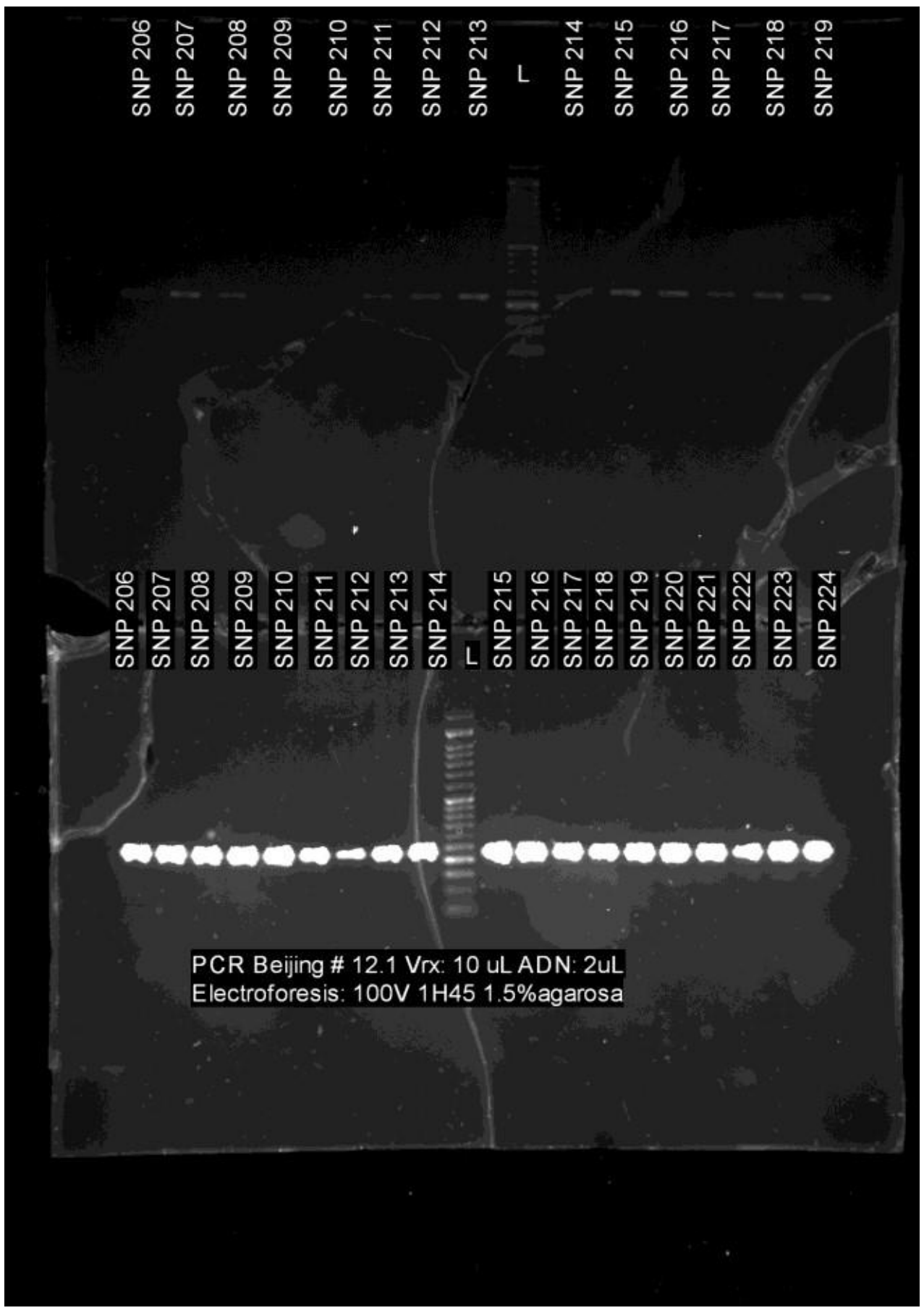

Gel image 12

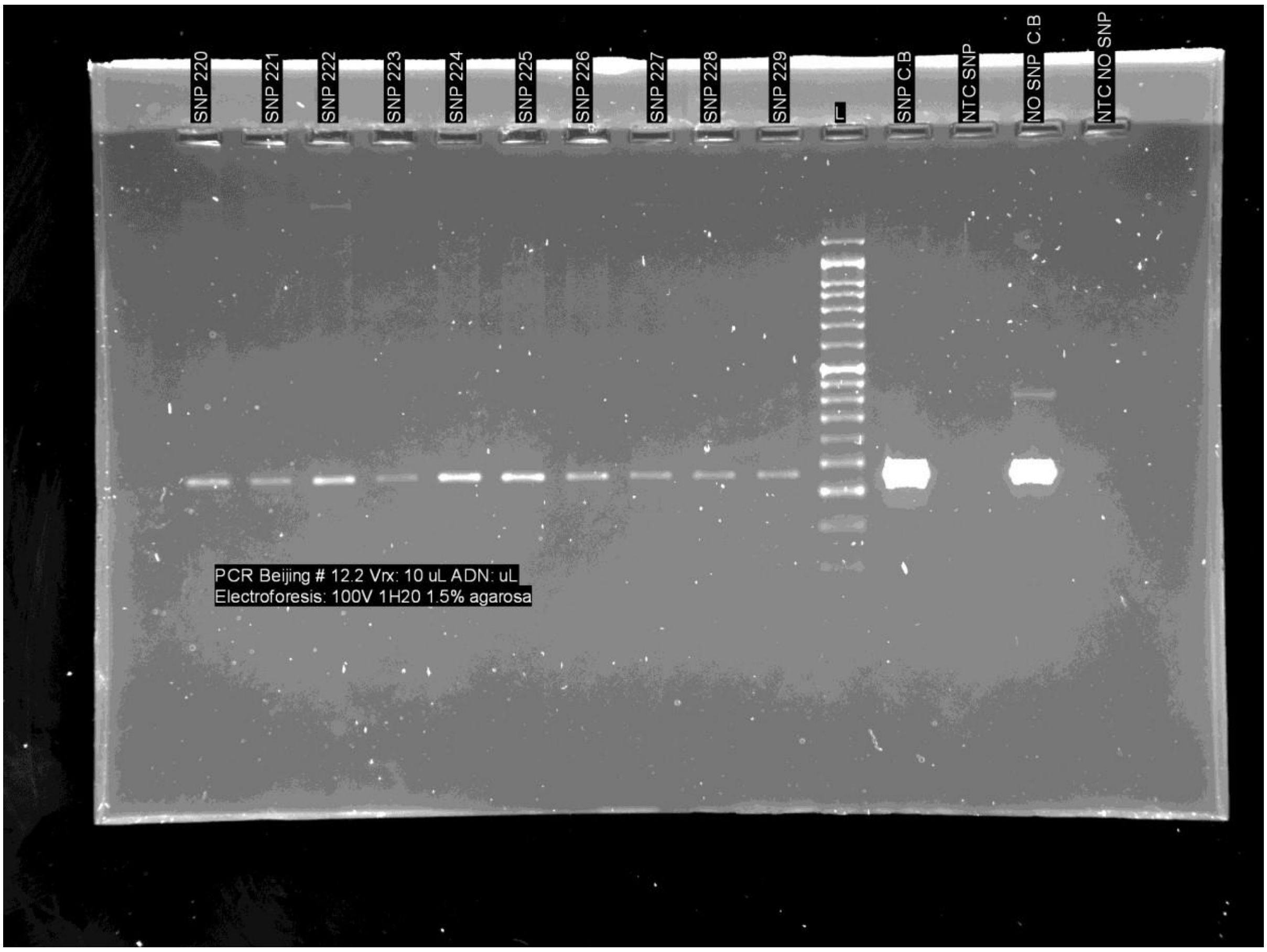

Gel image 13

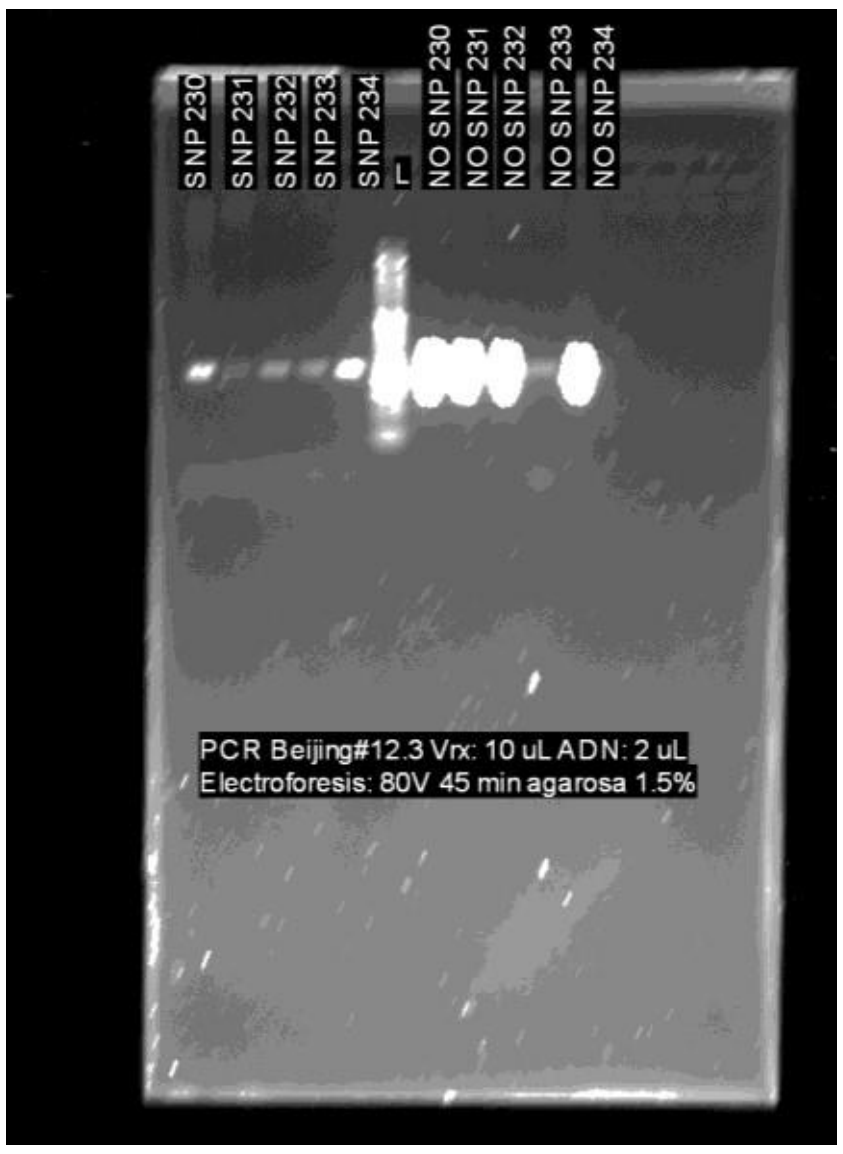

Gel image 14

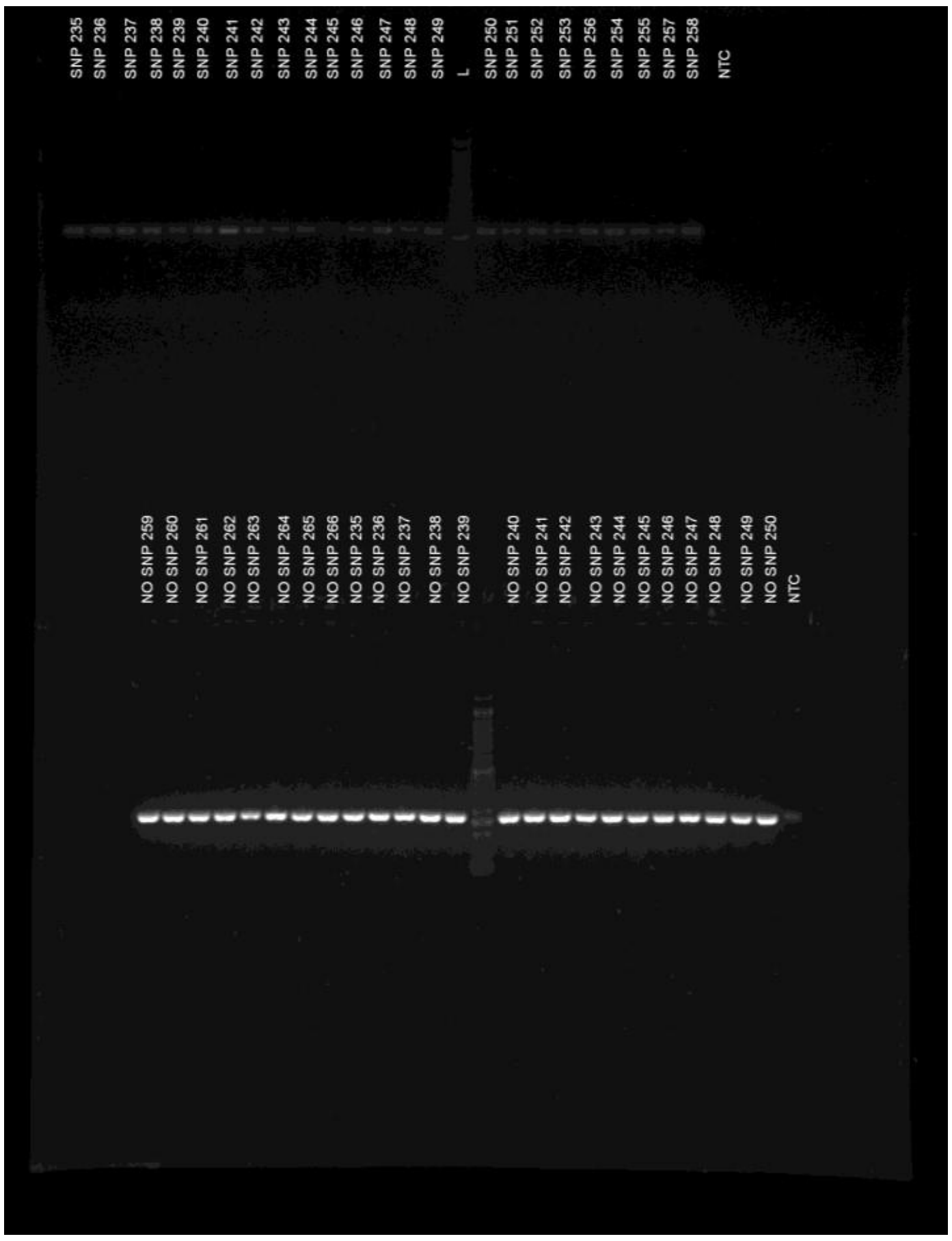

Gel image 15

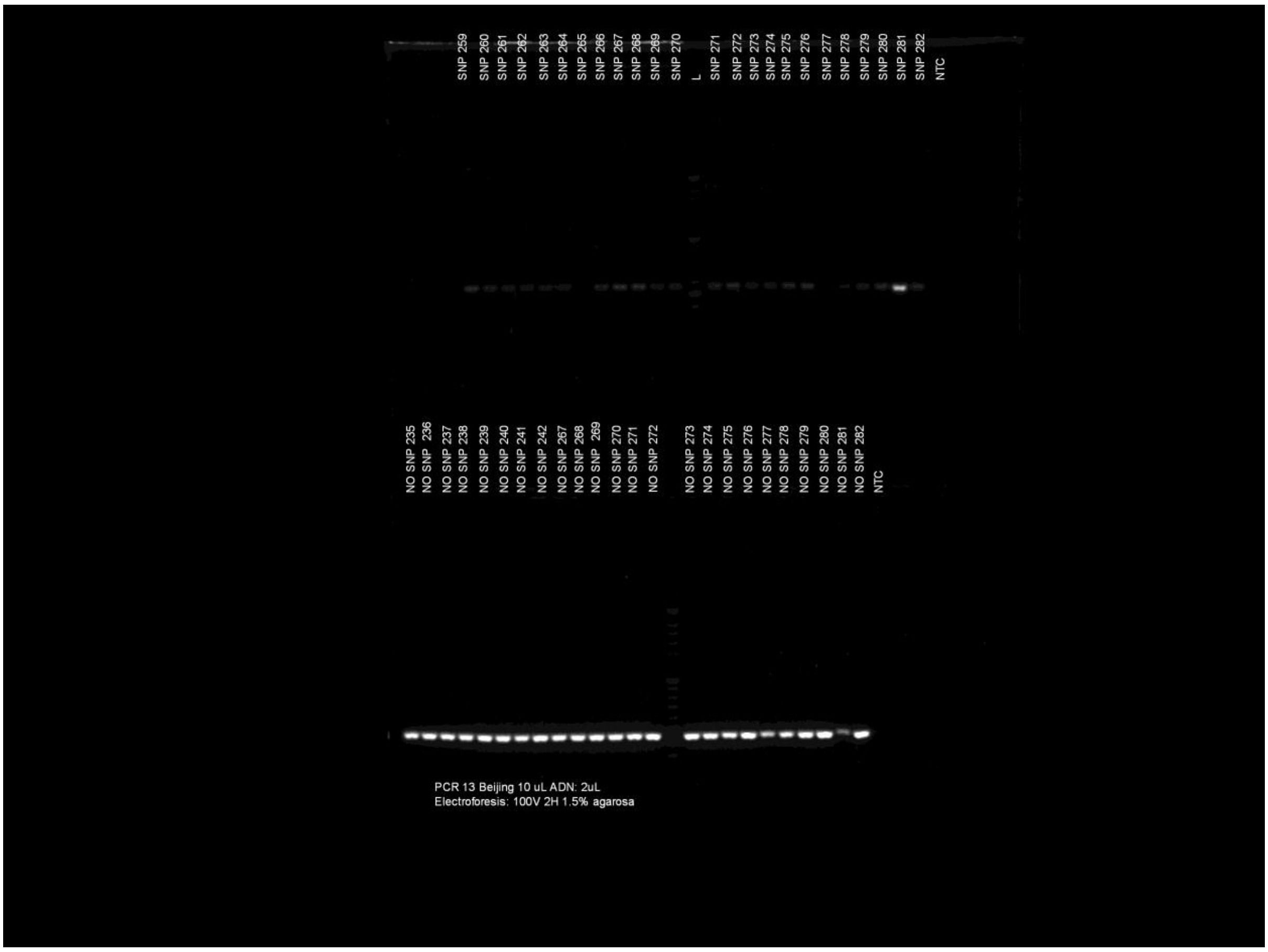

Gel image 16

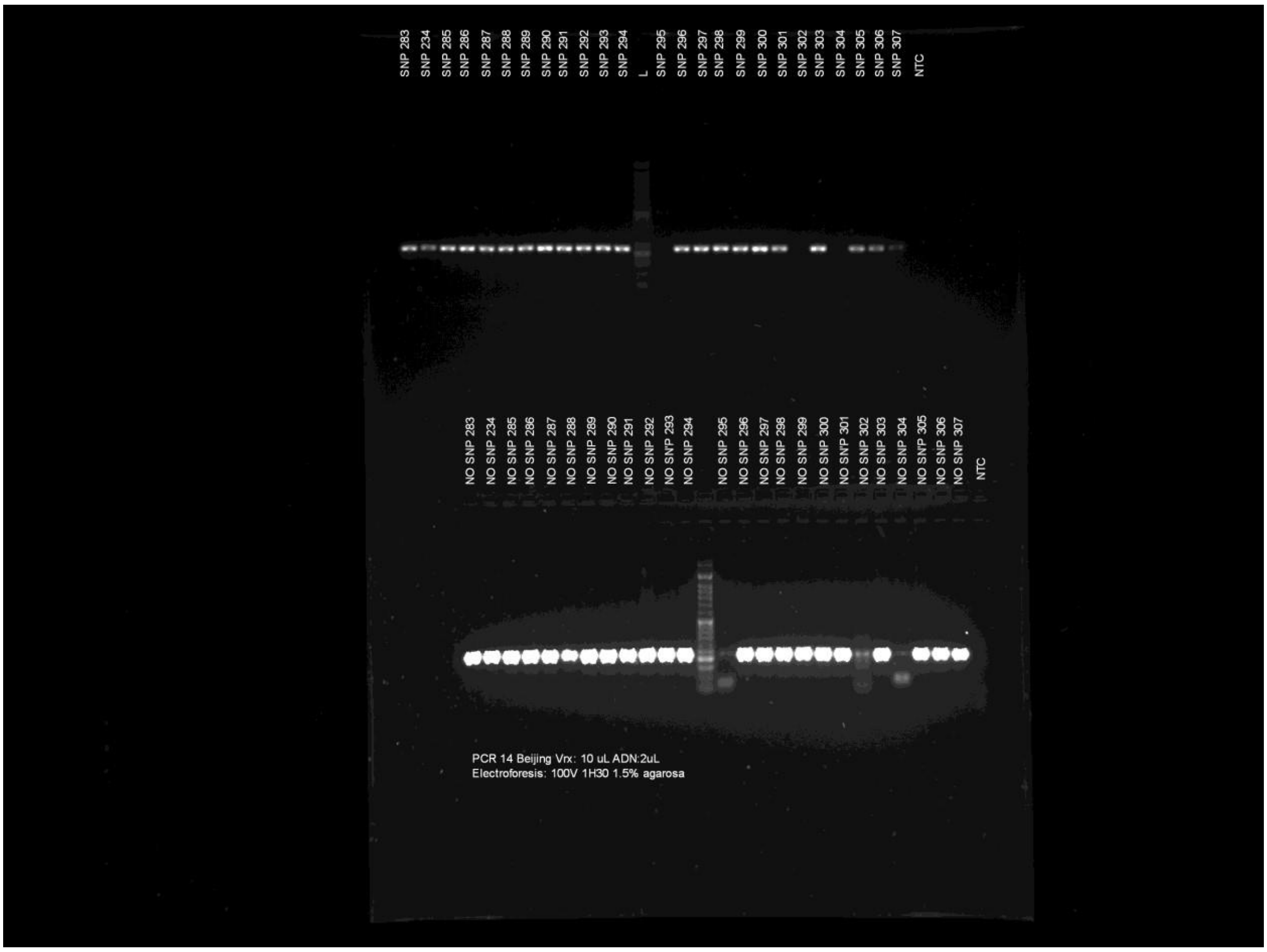

Gel image 17

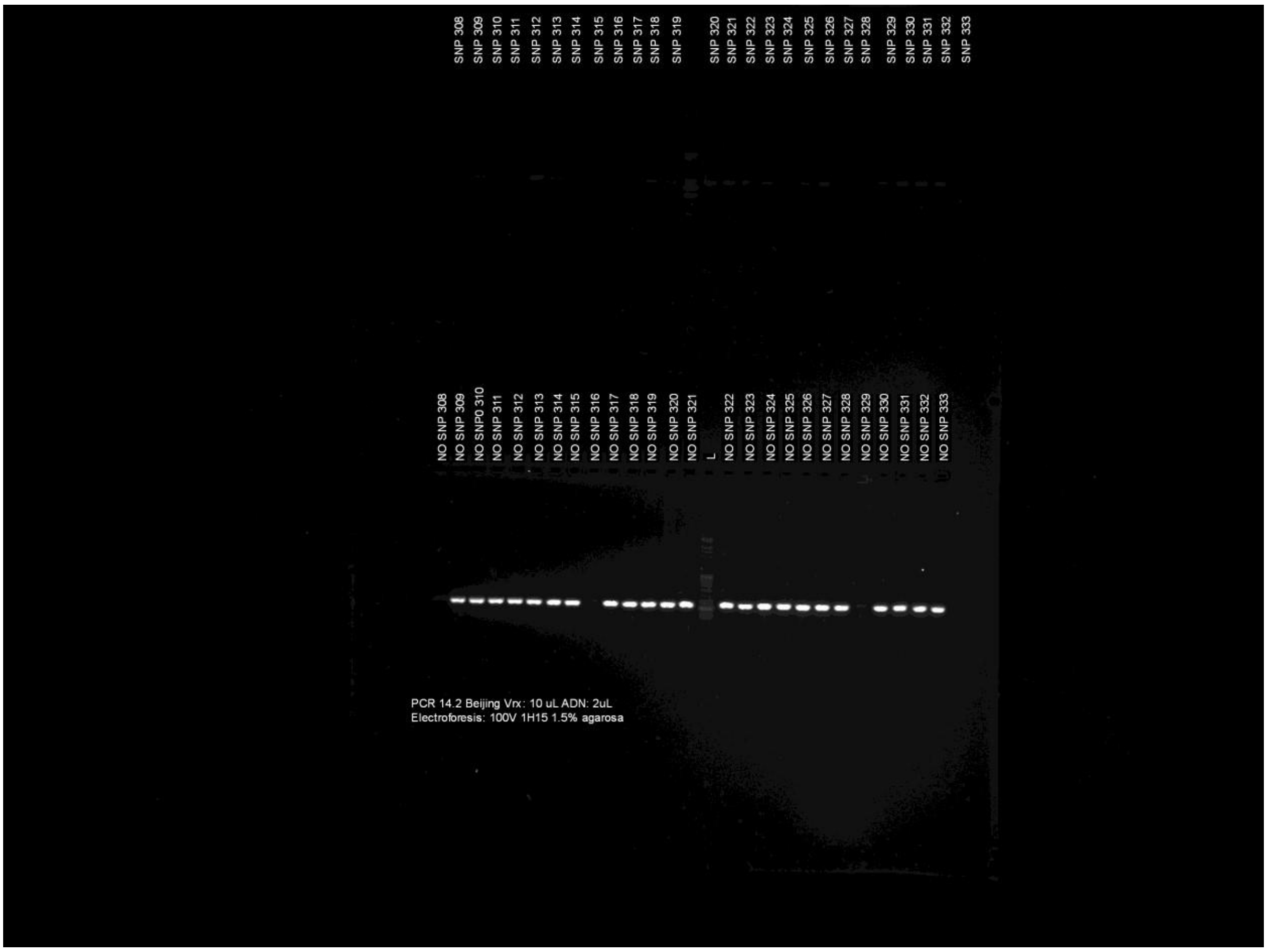

Gel image 18

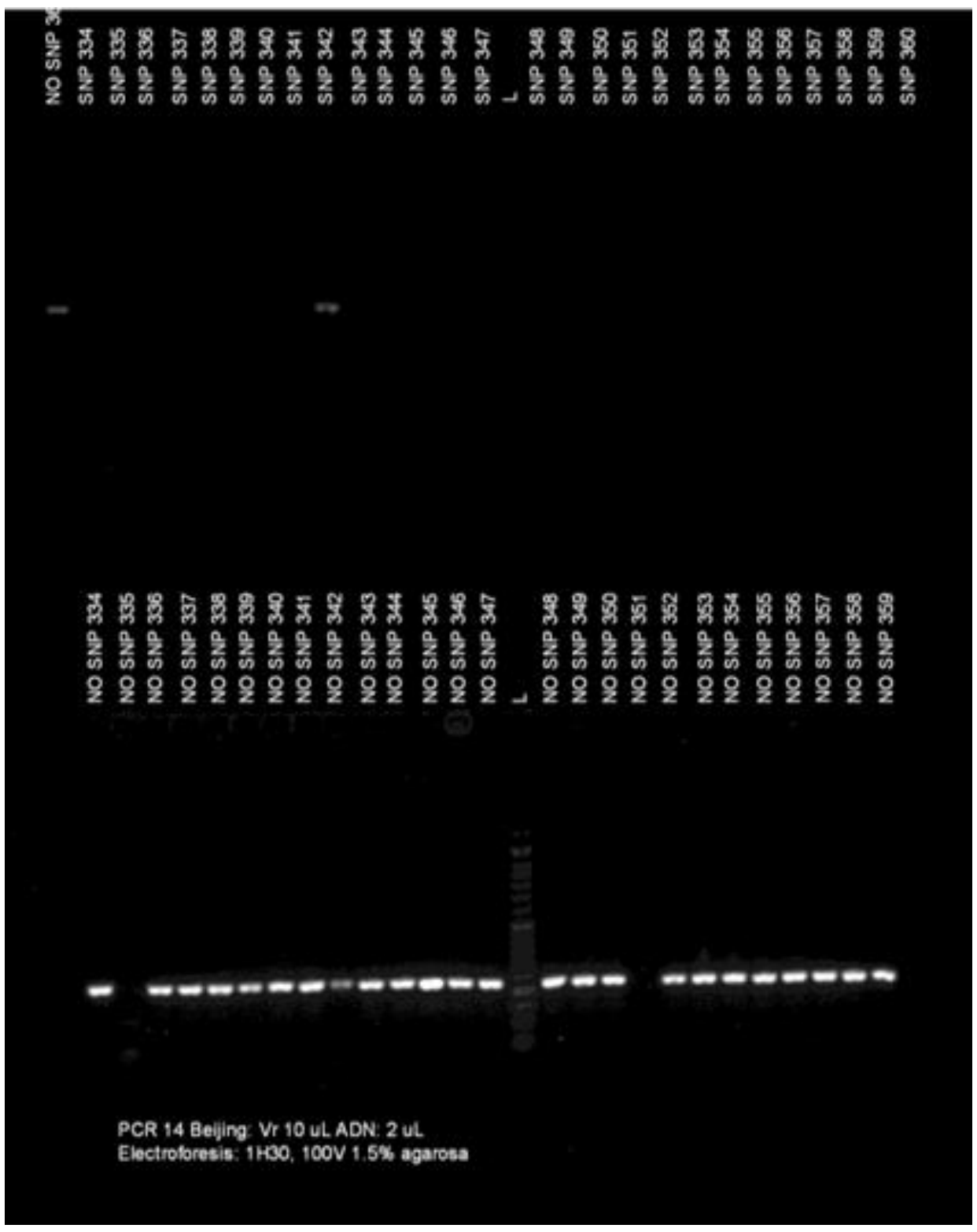

Gel image 19

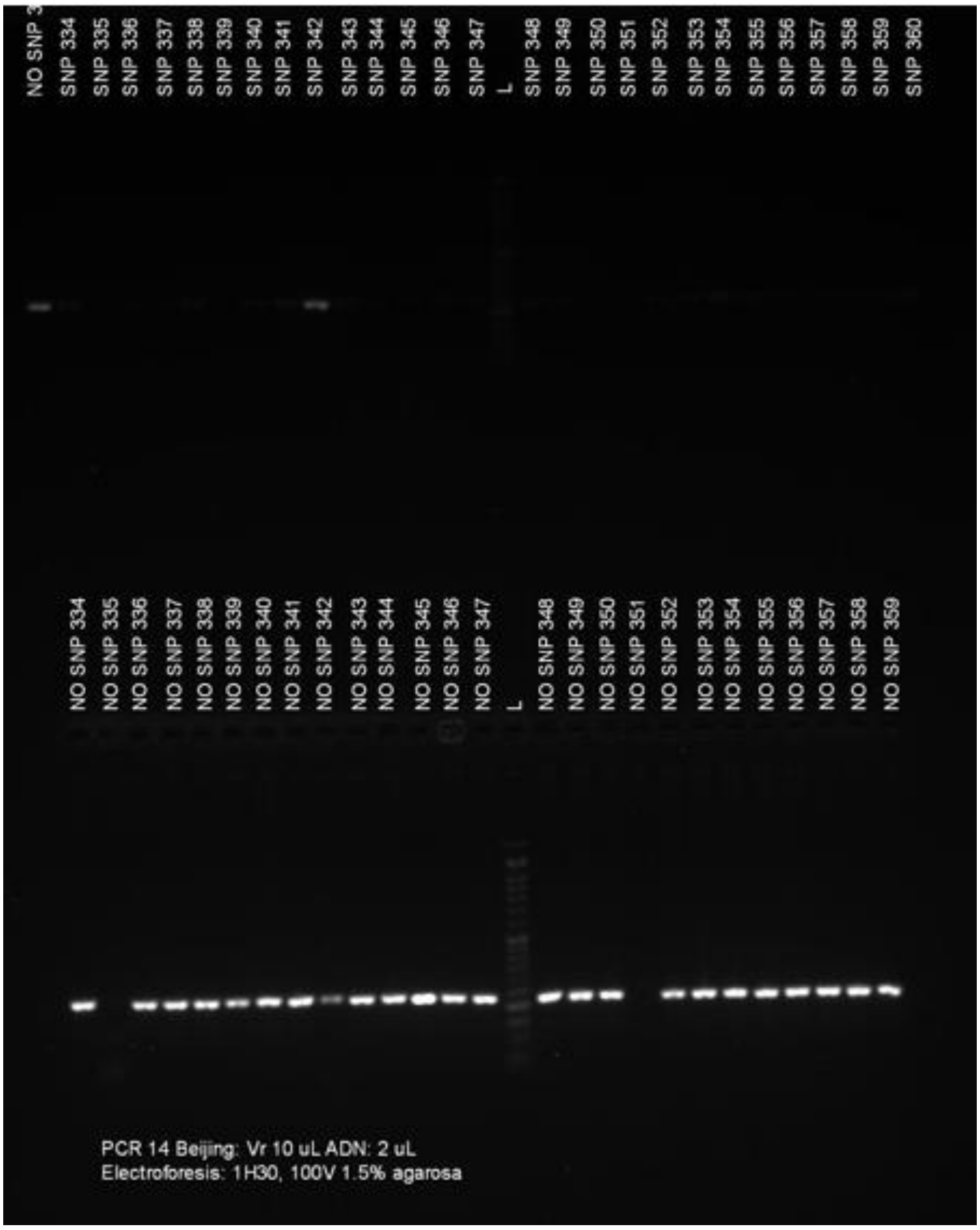

Gel image 20

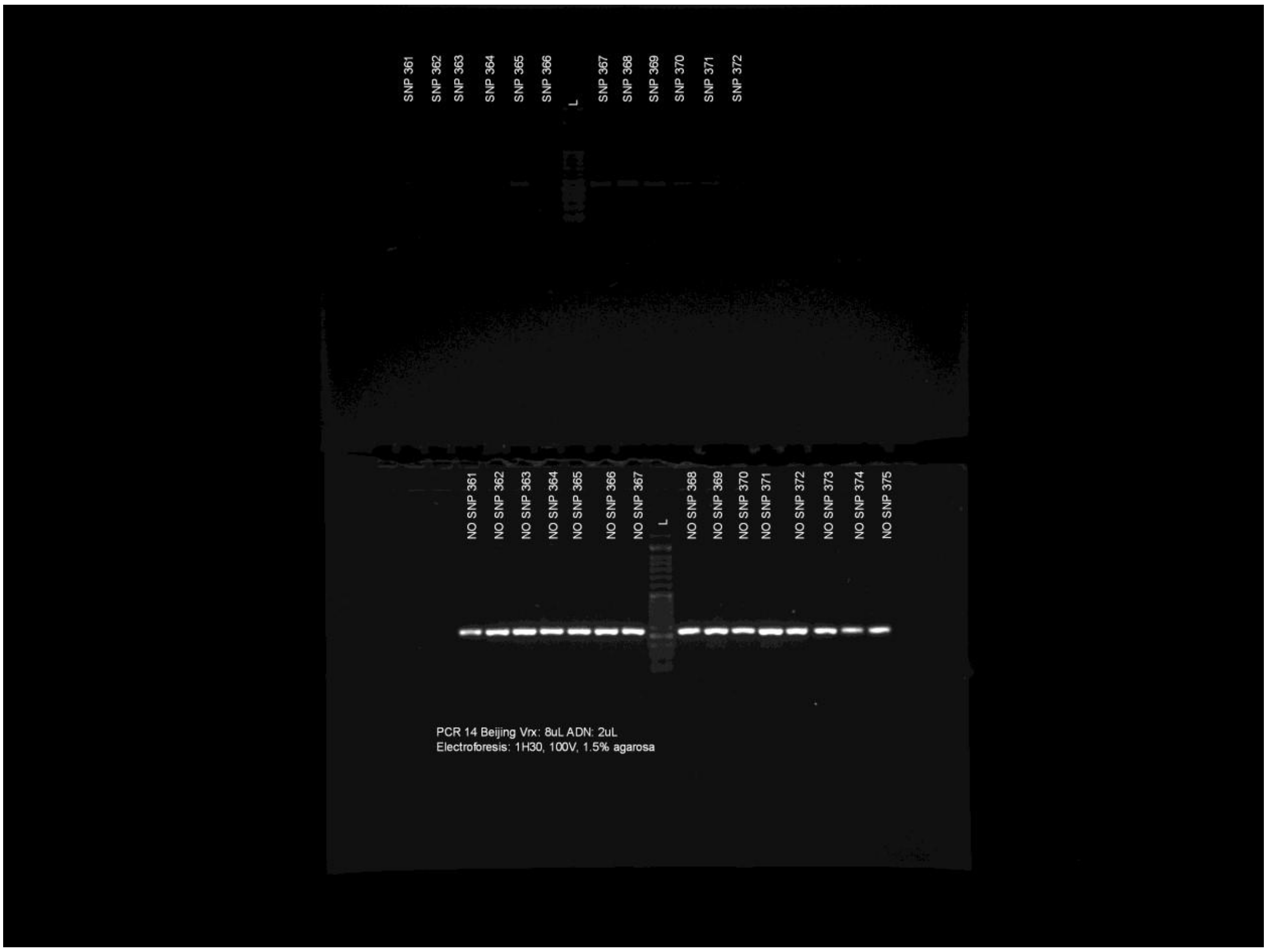

Gel image 21

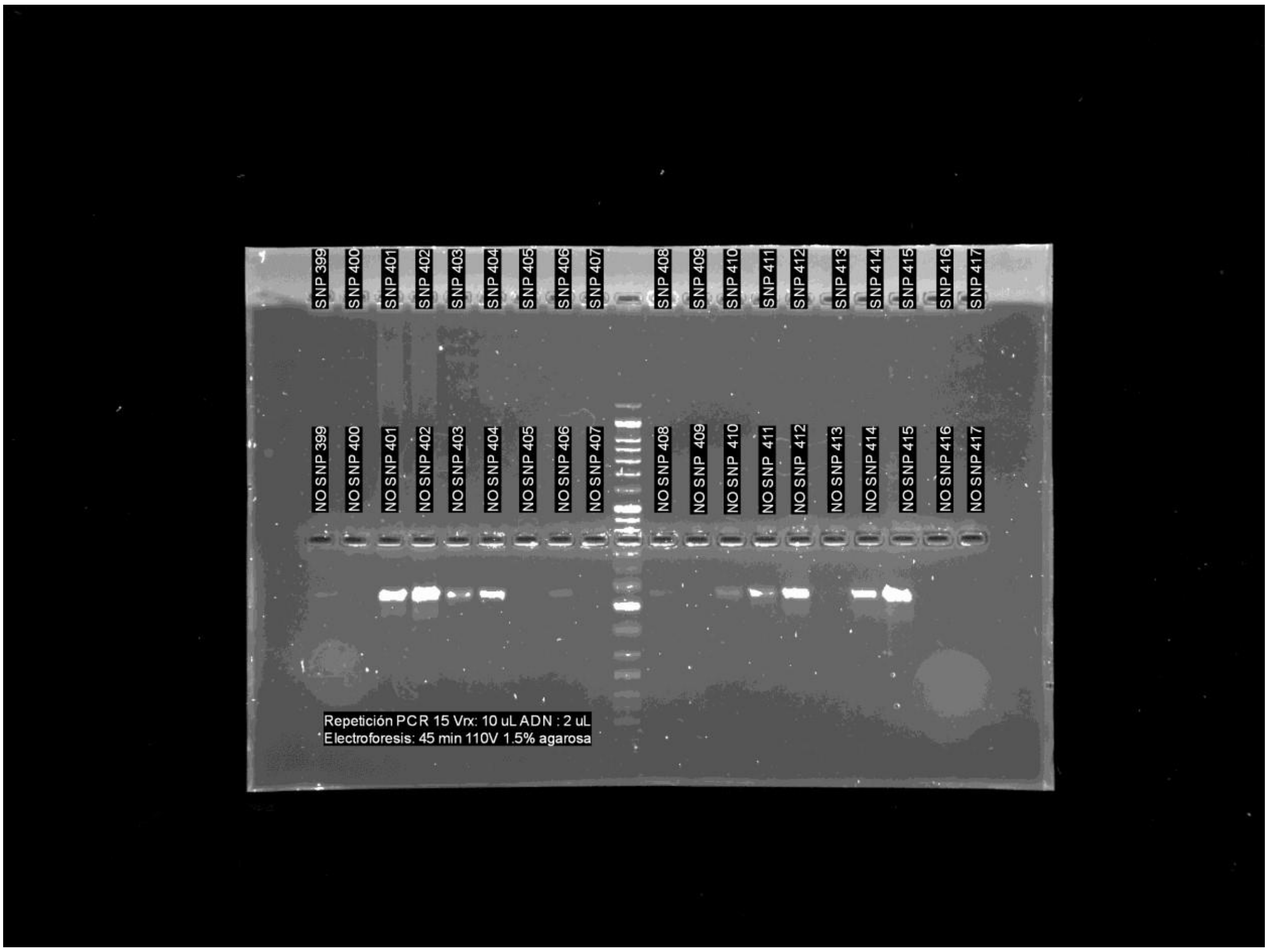

Gel image 22

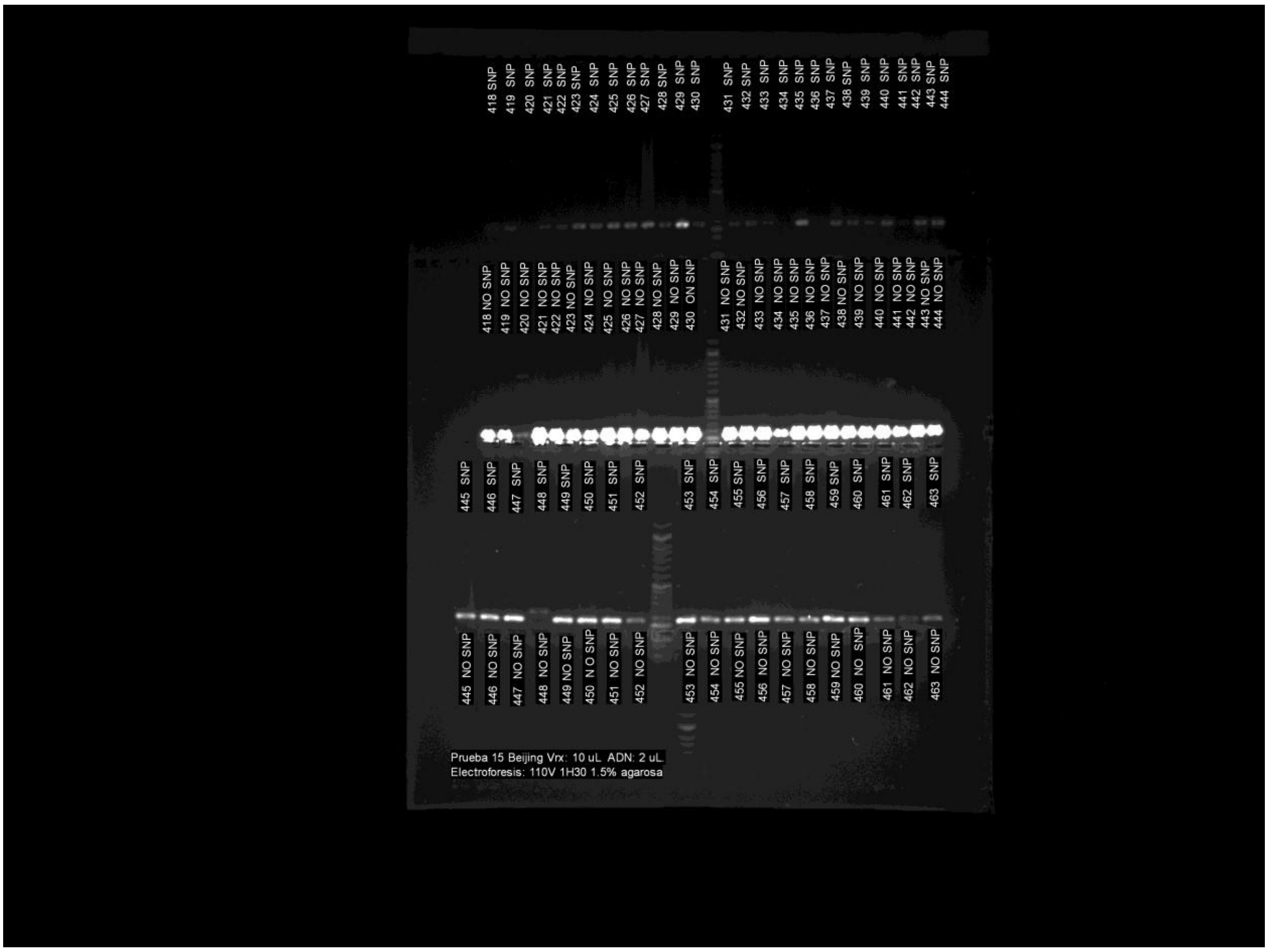

Gel image 23

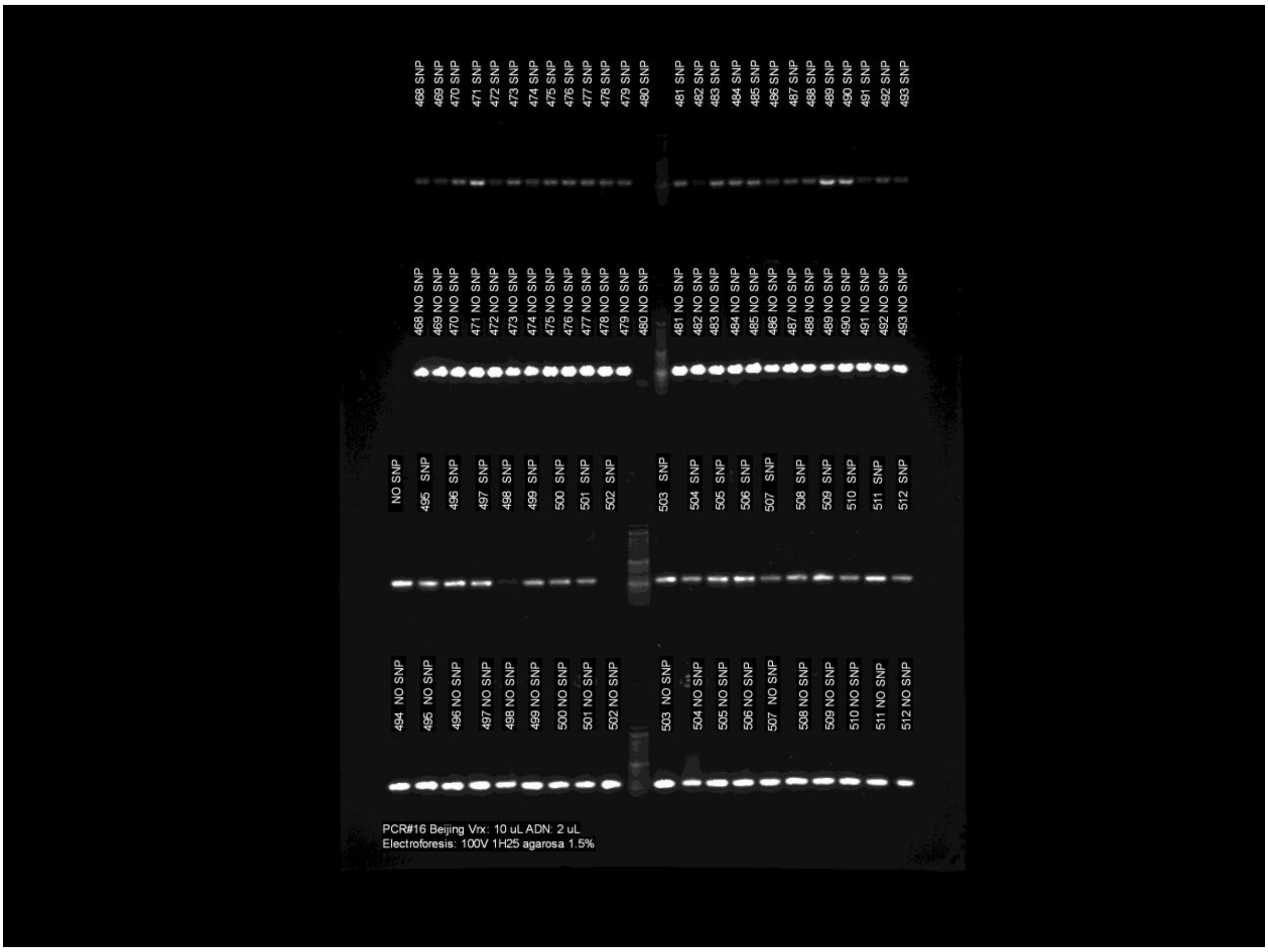

Gel image 24

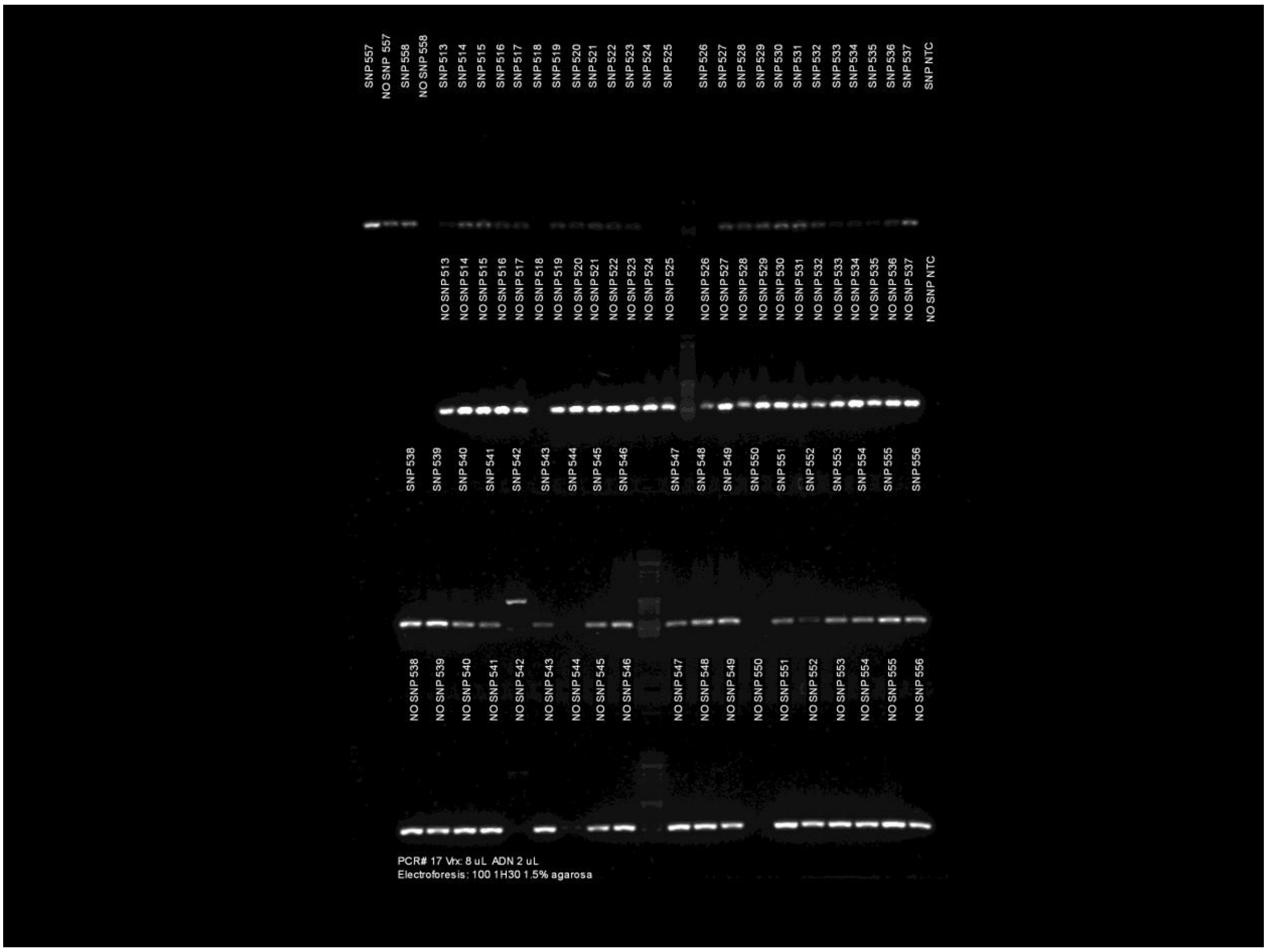

Gel image 25

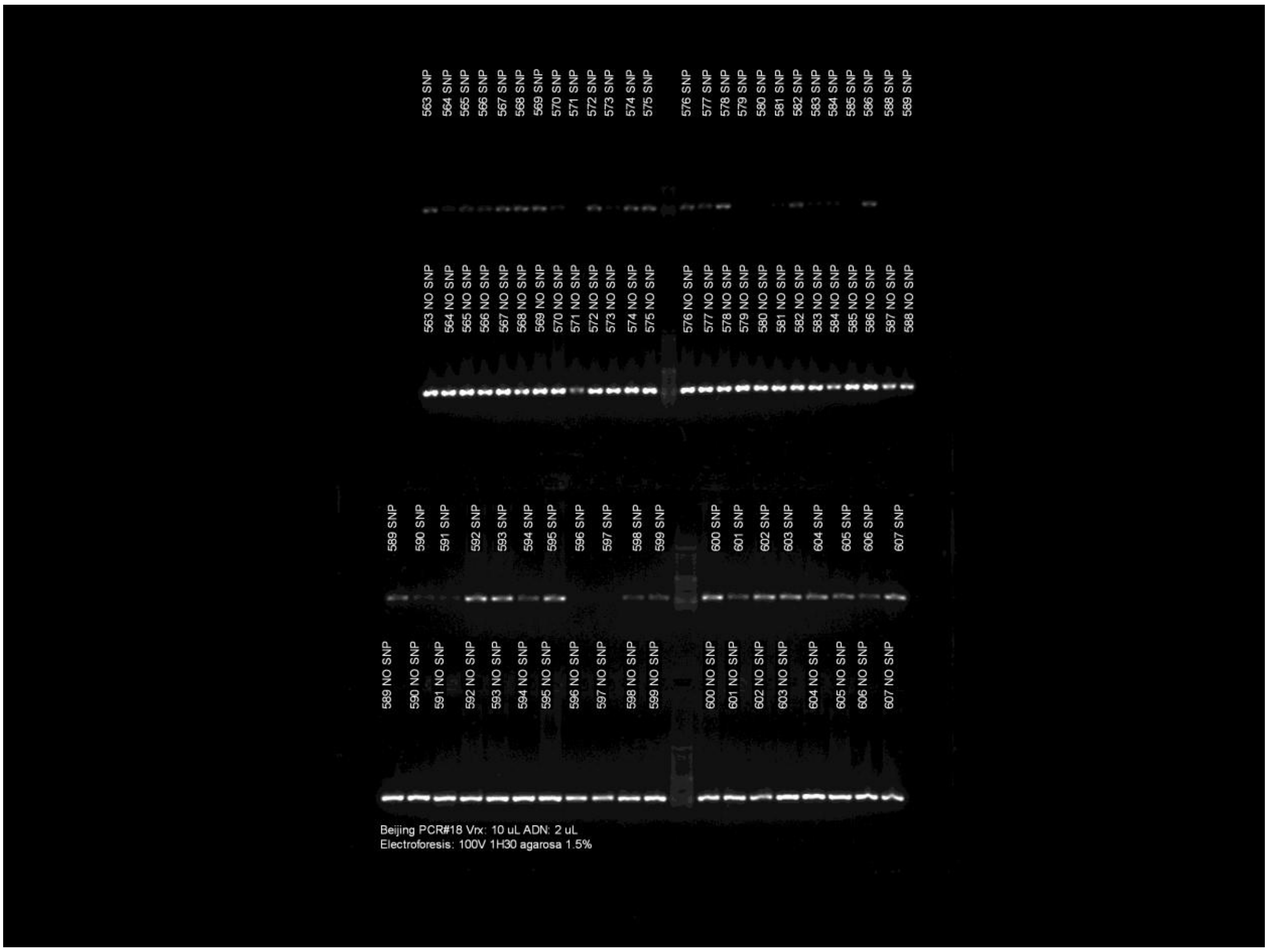

Gel image 26

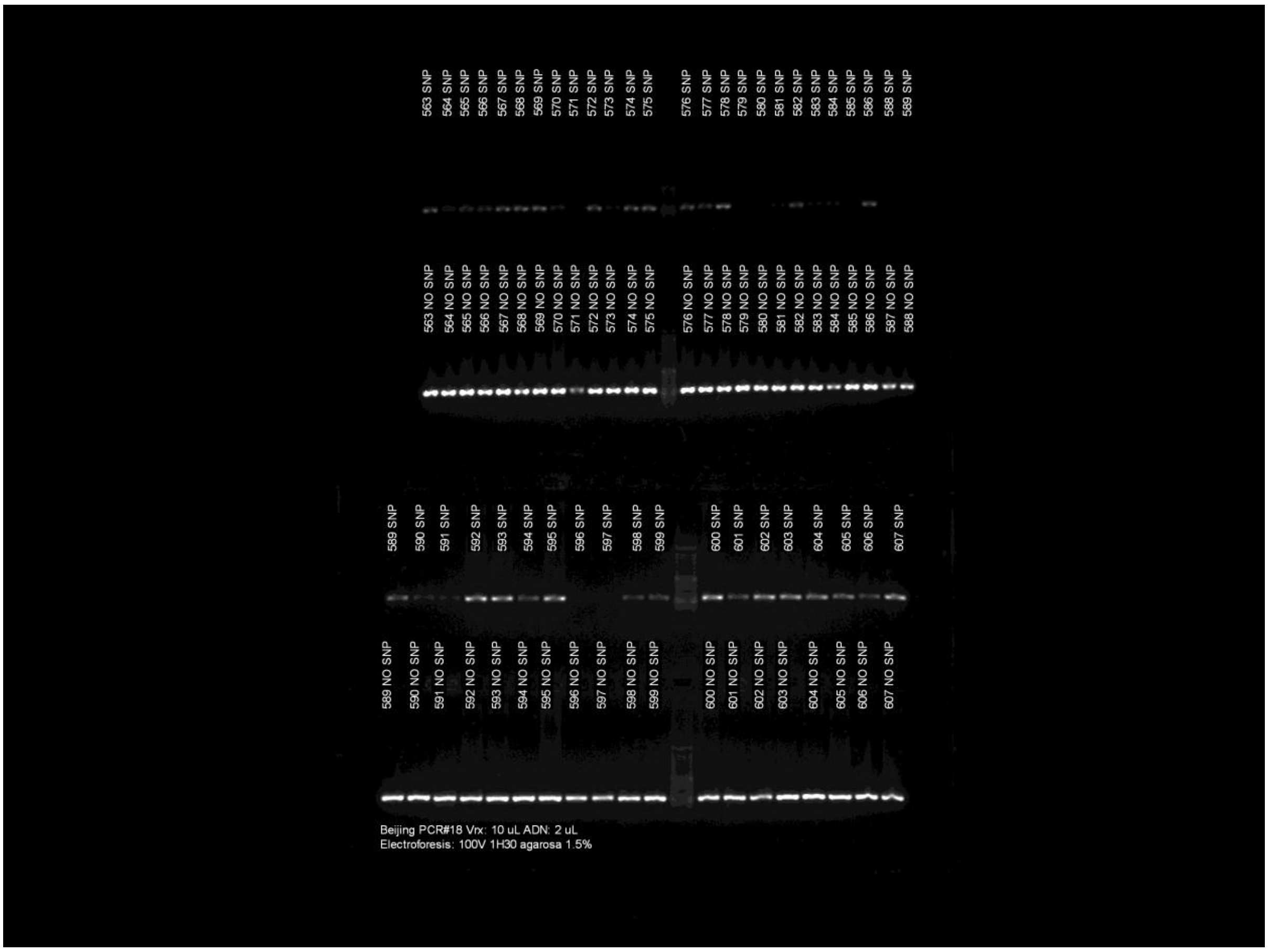

Gel image 27

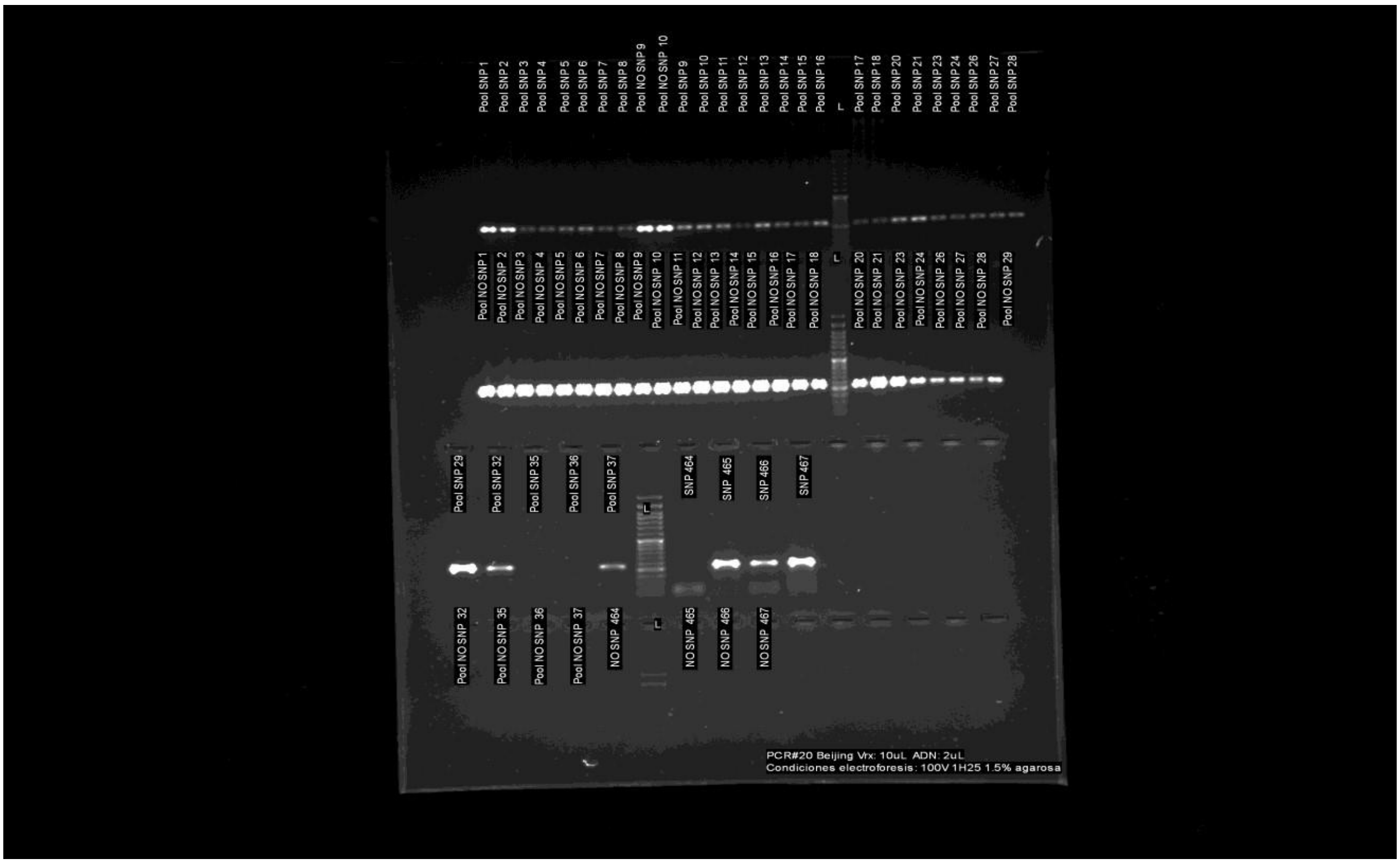

Supplement: Supplementary file 1 [file Data_Sheet_1.PDF]
