# Supplementary material for: Monitoring changes in the prevalence of the Beijing lineage of Mycobacterium tuberculosis in Ecuador: 2018 and 2023
Source: Front Public Health. 2026 Oct 27;14:1967382. doi: 10.3389/fpubh.2026.1967382 (PMC13748251; doi:10.3389/fpubh.2026.1967382)

Supplementary Figure S2. Spoligotyping membrane showing the hybridization pattern of the Beijing-positive *M. tuberculosis* isolate.

Spoligotyping was performed at the University of Extremadura (Spain) following the standard protocol. The membrane shows the hybridization patterns of the 43 spacer sequences of the Direct Repeat (DR) region, with isolates arranged in rows (numbered in red at the left) and spacers in columns. The isolate identified as Beijing in this study (sample ID Mach 1277; MYCO_025_Ago25) corresponds to row 8 [highlighted] and yielded the octal code 677777600060770. This spoligotype pattern did not match any registered pattern in the SITVIT/SpolDB4 database (assigned as "not identified"/orphan), and the MIRU-VNTRplus database classified it as Delhi/CAS; both assignments were discordant with the Beijing classification subsequently confirmed by whole-genome sequencing (sublineage L2.2.1). This discordance illustrates the limitations of spoligotyping for the accurate lineage assignment of this isolate. The remaining rows correspond to isolates from unrelated experiments processed on the same membrane and are not part of the present study.

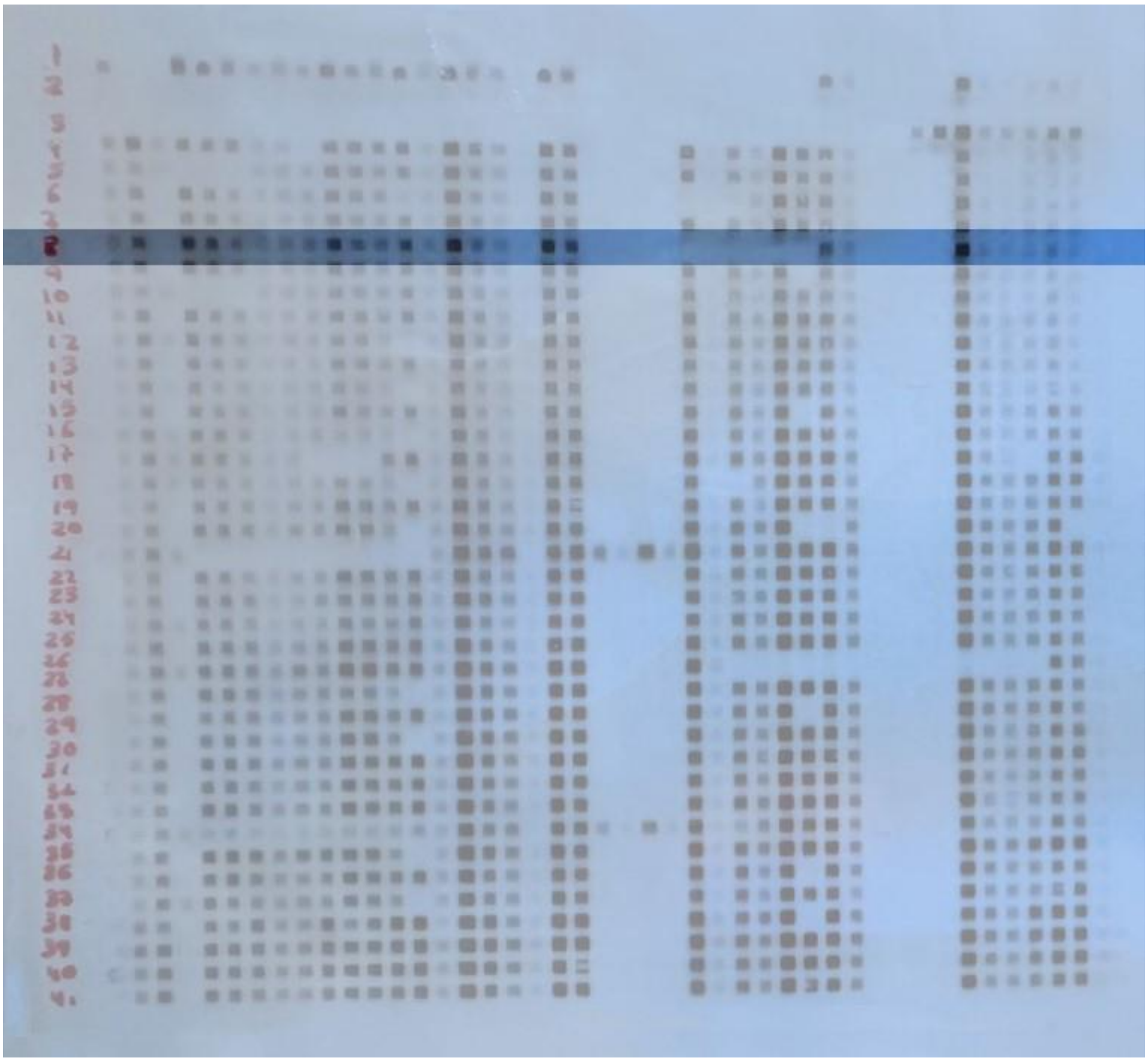

Supplement: Supplementary file 2 [file Data_Sheet_2.PDF]
